# Effects of Ga Substitution on the Local Structure of Na_2_Zn_2_TeO_6_

**DOI:** 10.1021/acs.inorgchem.2c01431

**Published:** 2022-08-09

**Authors:** Frida Sveen Hempel, Federico Bianchini, Bjørnar Arstad, Helmer Fjellvåg

**Affiliations:** † SINTEF Industry, Forskningsveien 1, Oslo 0373, Norway; ‡ Department of Chemistry and Center for Materials Science and Nanotechnology, University of Oslo, Oslo 0371, Norway

## Abstract

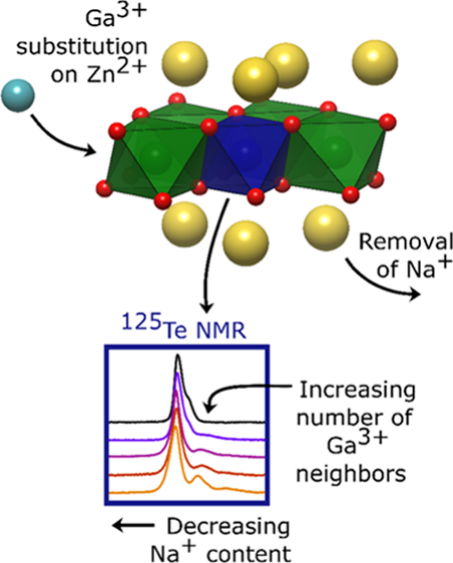

In the work presented here, we prepared Ga-substituted NZTO (Na_2–*x*_Zn_2–*x*_Ga*_x_*TeO_6_, *x* = 0.00, 0.05, 0.10, 0.15, 0.20) layered materials with a soft chemical, citric acid-based synthesis method and characterized these by means of X-ray diffraction (XRD), ^23^Na and ^125^Te NMR, and by density functional theory (DFT) modeling. The influence of randomly distributed Ga cations on the ^125^Te NMR spectra confirms the successful synthesis. With DFT-based linear response computations, we show that the local distribution of Na ions in the two neighboring interlayers influences the ^125^Te chemical shift, consistent with observations. DFT modeling suggests that some of the Na sites are rarely occupied in pure NZTO but become favorable upon Ga substitution. There are clear indications that Ga substitution gives an uneven distribution of Na ions in neighboring interlayers and that the Na structure in one layer affects the adjacent layers.

## Abstract 2

The influence of randomly distributed Ga cations on the 125Te NMR spectra confirms the successful synthesis.

## 1 Introduction

Layered oxide materials have been of interest for use in battery technology since the introduction of Li_*x*_CoO_2_ (LCO) as an early cathode material.^1^ While Li-ion batteries are currently dominating, Na-based technology is an interesting candidate for certain applications. This can be attributed to Na’s greater global distribution^2^ and the negligible weight difference in the larger picture of all battery components. Layered oxides may exhibit a negligible electronic conductivity while still having good ionic conductivity and may thus be candidates as solid-state electrolytes (SSEs). SSEs would replace the organic liquid electrolyte, which is highly flammable and can emit toxic gases in the case of fires,^3^ in addition to unwanted reactions with the electrodes.^4^ SSE materials should exhibit high ionic conductivity, low electron conductivity, and good stability^5^ and can be amorphous, polymeric or crystalline-like NASICON-types, certain sulfides, or Na-β-alumina.^6^

**Figure 1:**
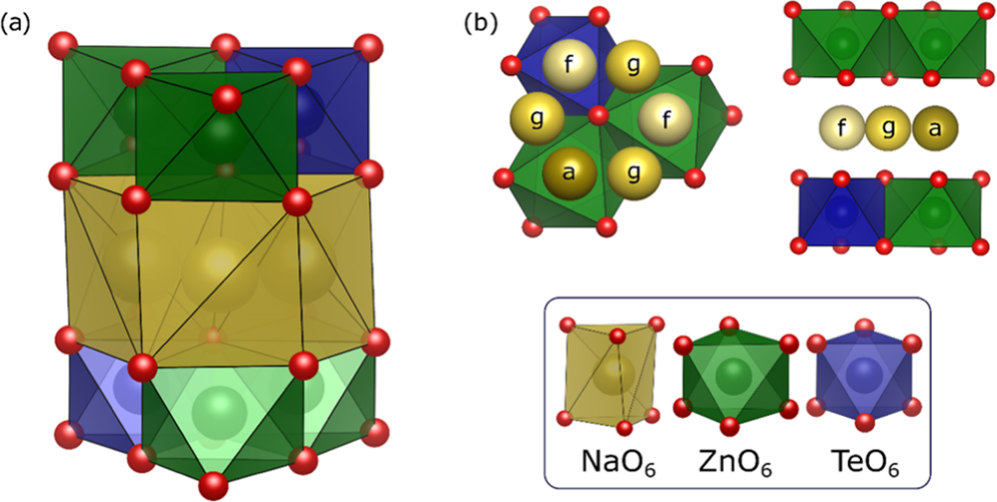
(a) Perspective view of the P2-type NZTO structure and space group *P*6_3_22. Coordination polyhedra of Te, Zn, and Na are shown (bottom). (b) Crystallographic Na positions, labeled as Wyckoff *a*-, *f*-, and *g*-sites. Note, on average, four out of 12 Na positions per unit cell are filled, and two adjacent sites cannot be simultaneously occupied due to distance constraints.

A structural classification for *A*_*x*_MO_2_ layered oxides was proposed by Delmas et al., with *A* being an alkali cation and one or several cations M located within (MO_2_)_*n*_ layers of edge-sharing octahedra.^7^ Depending on the stacking of the MO_2_ layers, the intercalated alkali ion may take octahedral (O) or prismatic (P) coordination. Furthermore, a number in combination with O or P refers to the number of distinct (MO_2_)_*n*_ layers in the stacking sequence, with two prominent stacking variants being the O3- and P2-types. An interesting family of quaternary layered oxides Na_2_M_2_TeO_6_ (M = Ni, Co, Zn, and Mg) was first described by Evstigneeva et al. in 2007^8^ and further characterized by Berthelot et al.^9^ These P2-type materials have 12 possible Na positions available in face-sharing trigonal prisms in each unit cell, although on average only four of them are filled with Na ions. The distance between adjacent Na sites is 1.7 Å, which is too short for the simultaneous occupation of Na ions. The M = Co, Ni members of Na_2_M_2_TeO_6_ are redox-active and are relevant as cathode materials,^10^ whereas M = Zn^11^ and Mg^12^ are candidates for solid-state electrolytes. The focus of our work has been on the Zn variant Na_2_Zn_2_TeO_6_ (NZTO) shown in Figure 1, which is in the space group *P*6_3_22 and has an alternating arrangement of the Te atoms across consecutive layers.^8^

This structure has three different crystallographic Na sites, with a multiplicity of three, two, and one in the interlayer gallery and below and above the layers of octahedrally coordinated Zn/Te. Hence, the central atoms of the octahedra have several possible local configurations with respect to the surrounding Na cations residing in the neighboring interlayers.

The three different Na prisms are differentiated by their coordination to the framework octahedra. A prism can either be positioned between two octahedra, where it will face-share its two ends with the octahedra on each side, or in the tetrahedral voids between the octahedra, where it is edge-sharing with the surrounding octahedra. The latter of these is the *g*-site, but the former is further differentiated by the cations in the framework layer: The *a*-site has a Zn octahedra on each side, and the *f*-site has one Zn octahedra on one side and one Te on the other. These sites are labeled using the symbols from the Wyckoff sites but omit the multiplicity. Any number in combination with *a, f*, and *g* denotes the number of filled sites instead, in line with our previous work on NZTO.^11,13^

Na_2_Zn_2_TeO_6_ shows Na conductivity, and by impedance spectroscopy, it has been reported to be 6.29 × 10^–4^ S cm^–1^. By introducing Ga at Zn sites, an increased conductivity of up to 1.1 × 10^–3^ S cm^–1^ has been reported.^14^ This substitution is reported to reduce the grain boundary resistance;^15^ however, Sau and Kumar et al. report that the main effect is decreased Na–Na repulsion due to the increased Na vacancy concentration.^16−18^ Karna et al. observed indications for Na ordering in a similar layered Na_2_Ni_2_TeO_6_ based on Fourier maps of synchrotron X-ray and neutron diffraction data.^19^ On the other hand, *ab initio* molecular dynamics studies of NZTO supported a disordered Na distribution.^13^

Solid-state synthesis is the most common synthesis method for the abovementioned layered oxides.^9,11,20−22^ This methodology is based on diffusion between and inside reactant/product grains, which makes the homogeneous substitution of elements in low concentrations challenging, as shown in previous reports, which indicate that the amount of unknown secondary phases increases with Ga substitution.^23^ The synthesis reaction pathway for the similar Na_x_CoO_2_ material is complex and depends on several factors and was reported by Bianchini et al. as an interplay between thermodynamically stable phases and nonequilibrium kinetic phases, with a multistage, compositionally unrestrained reaction pathway.^20^ To ensure homogeneous substitution, wet chemical sol–gel methods are preferable as they shorten the diffusion paths and mix easily to form a homogeneous distribution of precursors.

In this article, we present a detailed experimental and computational study of Ga-substituted NZTO materials. The motivation behind the work has been to investigate an alternative synthesis route, ensuring homogeneous substitution, combined with a characterization method for verification of the nominal substitution, which must also ensure that no regions are unsubstituted. We have applied the citric acid method, where all cations are presumably mixed at the atomic scale in the form of complexes and precipitated in a homogeneous gel during heat treatment. The substitution of Ga^3+^ for Zn^2+^, leading to Na_2–*x*_Zn_2–*x*_Ga_*x*_TeO_6_, modifies the sodium content in the interlayer galleries and may, in turn, affect the local Na substructure, within and between neighboring two-dimensional (2D) intergalleries, thereby impacting the environment of Zn/Ga and Te cations. Special attention is therefore given to Ga’s effect on the average and local structure. For this, ^23^Na and ^125^Te magic angle spinning (MAS) solid-state NMR spectroscopy was applied to obtain information on the local environment of Na and Te and changes induced by Ga substitution. The energetics of different interlayer Na configurations has been determined from structural optimization calculations using density functional theory (DFT). Furthermore, DFT linear response calculations of NMR chemical shifts are used to help analyze and interpret NMR spectra. The results obtained on the basis of high-quality samples made by the sol–gel method are discussed in relation to the existing literature on NZTO and related 2D materials.

## 2 Materials and Methods

### 2.1 Synthesis

Samples of Na_2–*x*_Zn_2–*x*_Ga_*x*_TeO_6_ with *x* = 0.00, 0.05, 0.10, 0.15, and 0.20 were synthesized using sol–gel synthesis. All precursors were added in relevant stoichiometric ratio, with the exception of Na_2_CO_3_, which was added in 10% excess to compensate for evaporation. The reactants ZnO (Sigma-Aldrich, 99.99%), Na_2_CO_3_ (Sigma-Aldrich, >99.5%), and TeO_2_ (Sigma-Aldrich, 99.995%) were dissolved by adding nitric acid (Sigma-Aldrich, 65%) using a magnetic stirrer on a hot plate at 50 °C until the solution became transparent. Subsequently, Ga(NO_3_)_3_·*x*H_2_O (Sigma-Aldrich, 99.9%) was dissolved in water and added. The level of hydration of the Ga-precursor was determined from thermogravimetric analysis (TGA). Finally, citric acid (Sigma-Aldrich, 99.5%) was added in a ratio of 5:1 with respect to the cations in the precursors.

The solution was heated to 180 °C while stirring to allow NO_*x*_ gas to evaporate, and after gel formation, the beaker was left overnight at 180 °C. The resulting powder was then heated to 450 °C for 12 h. The powder was ball-milled at 600 rpm for 20 min and pressed into pellets prior to final sintering reactions. The samples with *x* = 0–0.10 were sintered at 900 °C, while *x* = 0.15 and 0.20 at 800 °C, both using a heating/cooling rate of 5 °C. The top and bottom of the pellet were covered with the parent powder before sintering.

### 2.2 X-ray Diffraction (XRD)

Powder X-ray diffraction (XRD) data were measured on a Bruker D8-A25 diffractometer, using a Cu Kα_1_ radiation source and a Ge(111) Johansson monochromator and a Lynxeye detector. Data were collected for the 2θ range between 10 and 128°, using a step size of 0.005°, and samples were packed in a capillary for reducing preferred orientation. Rietveld refinement against the collected data was performed using Topas v6,^24^ and the background was fitted with a 10-term Chebychev polynomial.

### 2.3 Nuclear Magnetic Resonance (NMR) Spectroscopy

^23^Na (*I* = 3/2) and ^125^Te (*I* = 1/2) MAS NMR single transient spectra were collected at 11.74 T with a Bruker Avance AV III spectrometer using a 4 mm double-channel probe head at 295 K and at a MAS frequency of 10 kHz. The applied ^23^Na resonance frequency was 132.29 MHz, and 400 free induction decays (FIDs) after short pulses were accumulated for each spectrum. For the ^23^Na experiments, we used 1.5 μs excitation pulses at an RF-field of 33 kHz, calibrated using a 1 M NaCl(aq) solution. For ^23^Na, we measured, using the saturation recovery method, a *T*_1_ relaxation time constant of ca. 500 μs, and the recycle delay was set to 0.5 s. The applied ^125^Te resonance frequency was 157.79 MHz, and 2000 FIDs (NS) were accumulated for each spectrum using 90° pulses calibrated using a 1 M Te(OH)_6_ (aq) solution. Tests with variable recycle delays showed that the maximum intensity in the ^125^Te spectra were reached using a recycle delay of about 25 s. In addition, we measured with the saturation recovery method a *T*_1_ relaxation time constant of ca. 7 s for ^125^Te, and the recycle delay was set to 30 s. There were no observable differences between the various ^125^Te components in the spectra regarding relaxation rates.

The samples have been mainly stored in an Ar/desiccator after preparation/XRD analyses but have been some time in air since we have not worked in a completely inert atmosphere. ^1^H NMR was carried out to check hydroxyl groups and water level, and the samples showed a small broad peak that can be attributed to water that is slightly above the probe background level. The amount of hydroxyl groups was also only slightly above background levels. ^23^Na experiments with high-power proton decoupling gave identical spectra as those without decoupling. Hence, based on these data, we anticipate very small effects of water/OH groups on the samples, both in regard to structure, ion dynamics, and relaxation times. We also note that studies of water’s effect on similar materials show that the interlayer distance (*c*-axis) should increase with the intercalation of water.^25,26^ We do not see any sign of this in our XRD data, and hence we are confident that the water present is not of significance. Based on these points, we have decided not to work in a completely inert atmosphere with our materials.

The magnetic field was adjusted by setting the high-frequency peak of Adamantane to 38.48 ppm. In addition to the references 1 M NaCl (aq) for ^23^Na and Te(OH)_6_ (aq) for ^125^Te spectra, TeO_2_ (s) was used for comparison with chemical shifts calculated from DFT (*vide infra*). The shift values for aqueous ^23^Na and ^125^Te are set to 0 ppm. Before Fourier transform of the averaged FIDs, zero filling and apodization were applied to improve the line shape definitions and signal-to-noise ratio. The apodization was done by multiplying the FIDs with a decaying exponential window function with a processing line broadening (LB) factor of 250 Hz (^23^Na) and 50 Hz (^125^Te).

All NMR spectra were adjusted by proper signal phasing and baseline corrections. Curve fitting was performed using DMfit.^27^

### 2.4 Density Functional Theory Modeling

The DFT calculations were performed using the Vienna Ab initio Simulation package (VASP, version 5.4.4).^28−31^ The simulations build upon results and configurations obtained in our previous work^13^ and can be divided into two categories: (i) accurate calculations on a relatively small system to compute the Te chemical shift from linear response theory and (ii) AIMD simulations to compute the structural properties under Ga substitution. Structural optimization calculations were carried out to obtain reliable starting configurations compatible with the simulation parameters. All computations make use of the conjugate gradient algorithm.

The chemical shift for Te was evaluated by means of linear response theory using the method developed by Yates, Pickard, and Mauri.^32,33^ The shift was computed with respect to TeO_2_, which was then referenced back to Te(OH)_6_. Exchange and correlation were treated using the strongly constrained and appropriately normed (SCAN) semilocal density functional.^34^ The projector augmented wave (PAW) method was used to model core states. Additional technical details can be found in Section S1 in the Supporting Information (SI).

The Ga-substituted systems were modeled using a supercell structure of the configuration reported as the most stable in our previous work.^13^ A 3 × 2 × 1 supercell system (16.03 × 18.48 × 11.37 Å^3^) of said configuration was constructed, large enough to render the interactions between point defects and their periodic images negligible and to perform molecular dynamics simulation. The system contains 24 formula units (264 atoms).

The AIMD calculations were performed on the supercell model of the original NZTO structure and the two most stable substituted configurations. The stoichiometry of the systems is Na_48_Zn_48_Te_24_O_144_, Na_46_Zn_46_Ga_2_O_144_, and Na_44_Zn_44_Ga_4_O_144_. For brevity, the Ga-substituted systems are labeled as 2Ga and 4Ga, respectively. All AIMD simulations used a calculation setup similar to the one chosen to construct the initial configurations, with some minor modifications, as reported in Section S1.

AIMD simulations were performed both within the canonical (NVT) and the microcanonical (NVE) ensemble: the former for thermalization of the system and the latter for production. In both cases, we used the same parameters described in Section S1. Calculations within the canonical ensemble were modeled using the Nosé thermostat for controlling temperature oscillations.^35−37^ The Nosé mass parameter was set so that the period associated with these fluctuations was 40 fs. A time-step of 1 fs was found to be sufficiently small to avoid sudden jumps in the total energy of the system during the simulation. This setting was used to thermalize the system using the procedure described in Section S1.

Production calculations, performed in the microcanonical ensemble, compute 50 ps of dynamics. The analysis of the trajectories relies on three packages: MDANSE,^38^ the atomic simulation environment (ASE),^39,40^ and quippy, the python interface of QUIP.^41^ Additional software used in this work includes VESTA^42^ for the rendering of ball-and-stick and coordination polyhedra.

We would like to make some comments on the choice of methods when it comes to calculating ^125^Te NMR parameters from first-principles. Garaga et al. computed chemical shielding of various forms of TeO_2_^43^ using the methodology developed by Yates, Pickard, and Mauri.^32,33^ This work shows that meaningful results can be obtained already at the generalized gradient approximations (GGA) level using the Perdew–Burke–Ernzerhof (PBE) functional. Lizion et al. computed the NMR shift of Ge—Te structures including vacancies.^44^ As the large size of the systems considered and the presence of crystal defects render first-principles calculations particularly challenging, the authors opted for the Virtual Crystal Approximation, a less-demanding approach neglecting the distortion of the lattice due to vacancies and only considering one average site for the entire structure. Albeit not accurate, this approach allowed the identification of a large number of vacancies, which is correlated with higher chemical shift values. Furthermore, Alkan et al. showed that for ^125^Te, it is important to include relativistic effects.^45^ They used cluster models and showed that methods that included relativistic corrections had to be applied for accurately modeling the magnetic-shielding principal components of ^125^Te in a range of different compounds. This work also offers a comparison between results from different functionals, showing that, while hybrid functionals are required to obtain accurate results, GGA-level functionals do provide satisfactory results. Since Alkan et al. studied a range of different compounds and bonding types around Te, it was important to adopt methods that captured the essential parts of electronic structure and energies and magnetic and relativistic effects for heavy nuclei. However, in our case, we investigate very similar compounds and compare configurations that are quite similar both in structure and properties; hence, cancellation of errors is expected to occur. Even with the Ga incorporation, the closest Te surroundings are quite similar and only modulated slightly by the Na ions, and as long as we have found a functional that gives parameters close to experimental findings, other complicating effects will typically be canceled when making comparisons.

## 3 Results and Discussion

### 3.1 Crystal Structure

**Figure 2:**
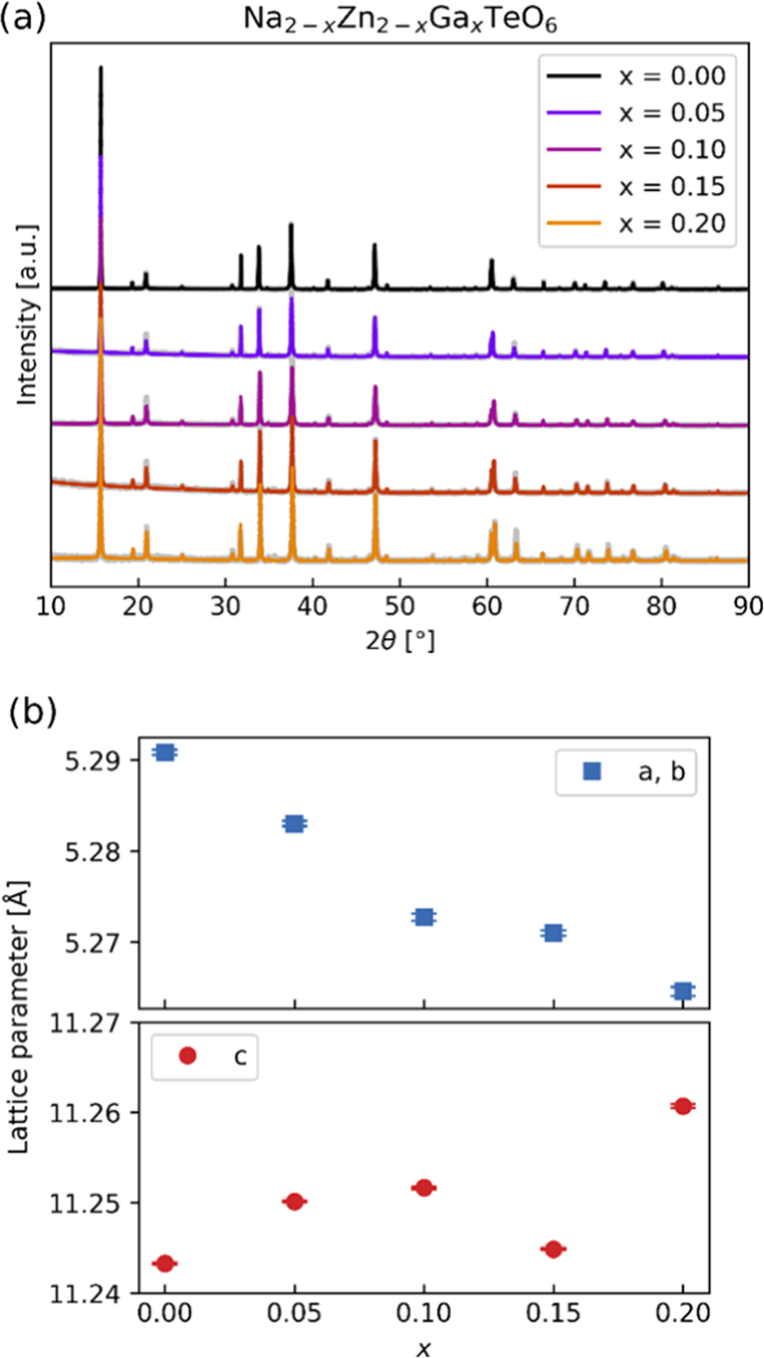
(a) Observed and calculated (Rietveld refinement) X-ray diffractograms of Na_2–*x*_Zn_2–*x*_Ga*_x_*TeO_6_, with increasing Ga concentration *x*; wavelength λ = 1.54 Å. (b) Variation in lattice parameters; space group *P*6_3_22. Error bars are calculated as standard error.

The diffraction data of Na_2–*x*_Zn_2–*x*_Ga_*x*_TeO_6_ are well described in the space group *P*6_3_22, which is consistent with other experiments.^11,14,15,22^ Diffractograms are shown in Figure 2a, with a more detailed Rietveld fit in Figure S1a and information in Table S1. The variation in unit cell dimensions with composition is shown in Figure 2b. Atomic coordinates and derived average bond lengths are listed in Tables S3, S4, respectively.

The SEM image of NZTO particles in Figure S2 shows that the material crystallizes as 1–2 μm thick platelets of some 2–5 μm in lateral size and with well-developed facets. This could give preferred orientation effects, even in the capillary setup. If the crystallites have an anisotropy similar to the particles, anisotropic size broadening could be expected, and adding this to the refinement improves the fit of the intensities. However, as shown in Table S2, either preferred orientation or anisotropic size broadening provided very low improvement in the *R*_wp_. This will be further explored in later work.

The elevated background in the 2θ range 16–24°, as shown in an inset in Figure S1b, has previously been ascribed to stacking faults.^32^ The two additional weak peaks indicated with blue arrows as *x* = 0.00 were earlier explained by the presence of an O’3 impurity,^22^ which, however, is unlikely for our samples owing to the lack of a shoulder/peak at 2θ = 16°. There is a small, unexplained peak at around 35° for all of the samples, which is tentatively ascribed to the Na substructure.^11,46^ A small ZnO impurity is fitted for sample *x* = 0.00 and may be 0.05, as shown in Figure S1c, but decreases for higher *x*-contents and is not visible in the patterns for *x* = 0.10, 0.15, and 0.20.

On increasing Ga substitution at the Zn site, the *c*-axis expands slightly. This may have different causes: cation repulsions due to higher charged gallium and O–O repulsion due to fewer Na cations or weaker Na–O bonding. The *ab*-plane contracts, leading to a decrease in the unit cell volume. The average bond lengths from the structure and the bond lengths from the DFT calculations described in Section 3.5 are listed in Table S4. In the average structure, the octahedral O-distances increase upon Ga insertion, but the DFT calculations confirm only the increase in the O–O distance in the Zn octahedra and show that prismatic O–O distances also increase instead of the expected decrease in the average model.

The *x* = 0.15 sample appears to have an anomalously short *c*-axis, with *c* and possibly *a* deviating from an otherwise clear trend. Repeated synthesis and analysis confirmed this result. As discussed in Section 3.3, we could confirm that the actual Ga/Zn ratio corresponds well to the nominal composition. This suggests that the reason for the anomaly might be rooted in the Na distribution, which has been reported to influence the interlayer distance.^47^ As discussed in Section 3.5, modeling reveals a preference for inhomogeneous Na distribution across the layers at high Ga substitution levels. Ga distribution was found to be homogeneous (*vide infra*), suggesting that the Na distribution alternates between layers to uphold charge neutrality. A series of different Na fillings, and therefore different layer distances, could sum up to different averages depending on the degree of filling and the filling/stacking sequence.

The previously reported Na site occupancies for NZTO vary considerably, especially with respect to the *f*/*g* ratio.^8,13,22^ The currently adopted site distribution is given in Table S3. Attempts to further refine the Na occupancies give no significant improvement in *R*_wp_, which probably reflects disorder, mobility, and weak scattering contrast. Note that the actual Ga distribution on Zn sites cannot be determined by XRD due to the low contrast between these neighboring elements in the periodic table, and hence nominal values are reported. To clarify aspects related to local ordering and the effect of Ga substitution, a series of NMR studies were conducted (see below).

### 3.2 ^23^Na NMR Study

**Figure 3:**
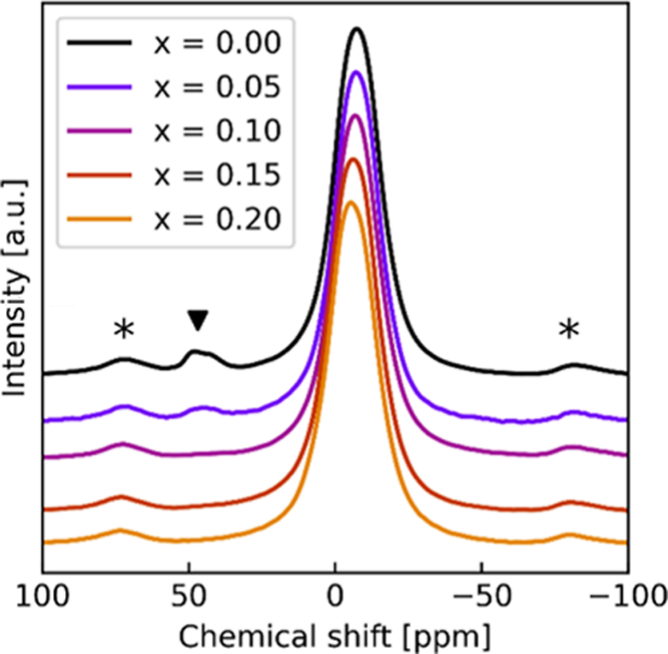
^23^Na NMR signals recorded for Na_2–*x*_Zn_2–*x*_Ga*_x_*TeO_6_. A peak of unknown origin is indicated with ▼. Spinning sidebands are denoted with *.

The ^23^Na MAS NMR spectra of the studied samples are shown in Figure 3. Curve fitting and superimposed spectra are shown in Section S3. Ideally, the sodium *a*-, *f*-, and *g*-sites with slightly different coordination environments should show up as closely positioned peaks, and one would expect that the prismatic coordination of Na should give an asymmetric environment with an appreciable electric field gradient (EFG) and hence peaks with typical second-order quadrupolar shape. However, the main peaks are quite similar for all samples (peak FWHM ∼ 2.3 kHz), and there are no signs in the line shape of strong quadrupolar couplings. With increasing Ga content, there is a slight shift of peaks to higher ppm values, and the small peaks around 45 ppm decrease and finally disappear. A small shift to higher frequencies may be due to a slight decrease in quadrupolar couplings; however, the low-frequency side (right side) does not change with increasing Ga content. The lack of observed features is interesting and may originate from several phenomena. Atomic disorder around the Na sites will result in a distribution of the chemical environments; however, distributions of quadrupolar couplings and/or chemical shifts will lead to an asymmetric peak shape with a tailing toward lower frequencies, which is not very obvious in our spectra.^48^ However, since NZTO and its Ga-substituted derivatives show Na ionic conductivity (*vide supra*),^14^ the lack of peak shape features could also indicate an exchange of Na cations at rates comparable to, or larger, than the spectral peak widths one would observe for a situation with completely rigid and static Na ions. Increasingly faster jump rates between various sites of prismatic coordination will average the quadrupolar couplings and chemical shift differences until a single peak is observed. To observe such a development, one would have to carry out variable temperature experiments cooling down to such a low temperature that Na ion dynamics were absent and compare with experiments at higher temperatures. The apparent symmetry of the main peak in the stacked plot (Figure 3), is slightly misleading. Fitting shows that the peak cannot be decomposed into one Gaussian/Lorentzian peak for any sample, and hence residual quadrupolar couplings likely remain. For layered Na_x_CoO_2_, Carlier et al. found that the ^23^Na signal was not fully averaged at room temperature and could only be fitted as one single peak first at 475 K.^47^

With increased Ga substitution, the peaks at 45 ppm disappear. Since they are located at higher frequencies than the main peak, they probably originate from Na at a lower coordination as this is typical for ^27^Al and also indicated for ^23^Na.^48,49^ The peaks around 45 ppm could be intrinsic to NZTO or due to a secondary phase. From tabulated values of chemical shift values for Na compounds, no obvious feature appears to explain the peaks.^49^ However, upon heating to 200 °C (addressed in another work to be published), the peak at 45 ppm disappears and the main peak shifts slightly to the left. However, the peak reappears upon cooling, suggesting some dynamic effects that exclude a secondary phase.

The integral of the 45 ppm peaks corresponds to about 3% of the total peak areas for pure NZTO. Hence, it is unlikely that the peak is due to the Na situated at a grain boundary, interface, or surface. In these cases, a high surface-to-volume ratio is necessary; however, this is not supported by any broadening of XRD peaks. We consider it unlikely that the peak may emerge from a substitutional defect of Na on either the Zn or Te position, due to the difference in charge and size. Under all circumstances, such a defect is expected to have a chemical shift to the right of the prismatically coordinated Na, considering that Na on the Zn site will be octahedrally coordinated. Due to the ambiguous decomposition of the ^23^Na MAS NMR spectra, the changes in the Na sublattice from the Ga substitution cannot be described in sufficient detail.

### 3.3 ^125^Te NMR Studies

**Figure 4:**
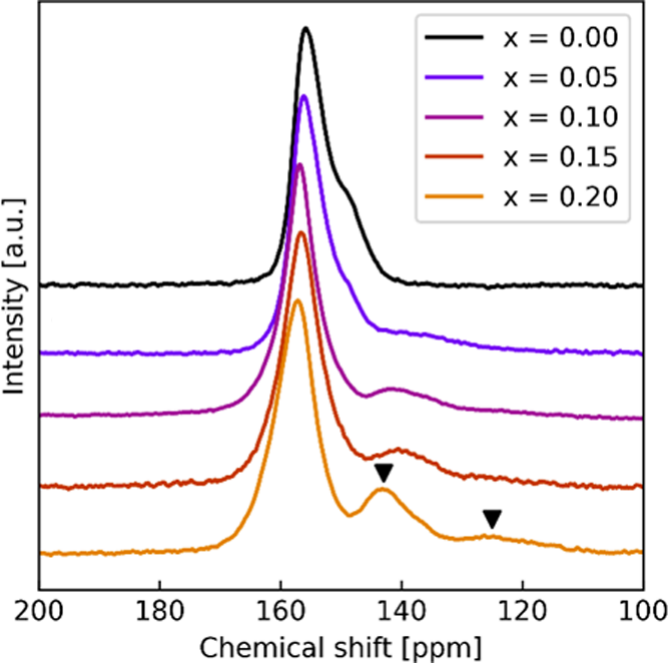
^125^Te MAS NMR spectra of Na_2–*x*_Zn_2–*x*_Ga*_x_*TeO_6_. Two peaks that appear at higher substitution levels are marked with black arrows for *x* = 0.20.

The NZTO-based materials were further characterized by means of ^125^Te MAS NMR; see spectra in Figures 4 and S4 for curve fittings. The ^125^Te spectrum of the NZTO (*x* = 0.00, black line, Figure 4) shows one peak at 155 ppm with a distinct shoulder at approximately 145 ppm. The physical origin of this shoulder feature will be discussed in Section 3.5. Integration of this shoulder feature (145 ppm) in the samples *x* = 0.00 and 0.05 is counted as part of the main peak (150 ppm) in this section. Furthermore, note that the spectrum cannot be unambiguously decomposed, and at least three Gaussian peaks are needed for a good peak shape description. As Te takes one single crystallographic site (Wyckoff site 2*c*) and is a spin 1/2 nucleus, the presence of multiple peaks must be due to different local structures around Te atoms. Upon Ga substitution, additional peaks emerge in the region below 140 ppm (Figure 4). All Ga-substituted samples show a feature around 140 ppm, while *x* = 0.15 and 0.20 show a weaker peak at about 125 ppm as well. Based on charge and size arguments, we claim that Ga substitutes for Zn are in compliance with the intensities of the XRD patterns. A Ga atom at a Zn site will therefore be within the second coordination sphere of three Te atoms. By curve fitting and integration, we estimated the relative amounts of the main peak at 155 ppm and the two side peaks at 140 and 125 ppm (see Table 1 below). The integration shows that the theoretical estimate of the number of Te atoms with different surroundings corresponds very well with the integrated ratio of the observed peaks, assuming the peak at 140 ppm to reflect Te atoms with one Ga neighbor and the 125 ppm peak to reflect two neighbors.

**Table 1:** Integrated Peak Intensities and Percentage of Te Atoms with at Least One Close Neighbor Ga Atom for Na_2–*x*_Zn_2–*x*_Ga_*x*_TeO_6_^a^

*x*	theoretical Te with at least one Ga neighbor [%]	*I* (155 ppm) [%]	*I* (140 ppm) [%]	*I* (125 ppm) [%]	sum small peaks [%]
0.00	0.0	100.0	0.0	0.0	0.0
0.05	7.5	86.4	11.1	0.0	11.1
0.10	15.0	83.9	16.1	0.0	16.1
0.15	22.5	75.0	22.4	2.6	25.0
0.20	30.0	71.2	22.3	6.6	28.9

a For *x* = 0.20, 10% of Zn atoms are replaced by Ga. Each Zn site has three Te neighbors, implying that 30% of the Te atoms for *x* = 0.20 have at least one Ga neighbor.

The highest substitution level of *x* = 0.20 should influence 30% of the Te atoms. The integration shows that the main peak amounts to 71.2%, and the two smaller peaks sum up to 28.8%, which is in good agreement with the expected value. An exception is *x* = 0.05, for which the integrated value is higher than the theoretical value (11.1 vs 7.5, respectively), which can be attributed to uncertainties in the integration of the weak peak at 140 ppm and the high influence of variations during phasing. The increase in only the two-neighbor peak (at 125 ppm) between *x* = 0.15 and 0.20 are indications that the Ga distribution is less random at high substitution levels. We note a change in the peak shape also for the main peak with increasing *x*. Hence, Te atoms with no neighboring Ga atoms are also affected. The nature of the changes is explored in Section 3.4. Note that there are no indications that any ^23^Na or ^125^Te peaks are due to significant amounts of a secondary phase. Based on our NMR analyses, it is likely that samples correspond to the nominal composition targeted in the synthesis, except for maybe the material with *x* = 0.05. This suggests that the deviation in the *c*-parameter for the *x* = 0.15 sample is not due to aspects of Ga concentration. Furthermore, the NMR data clearly indicate that the homogeneous substitution of Ga is obtained upon sol–gel synthesis.

### 3.4 DFT Calculations of ^125^Te Chemical Shifts

**Figure 5:**
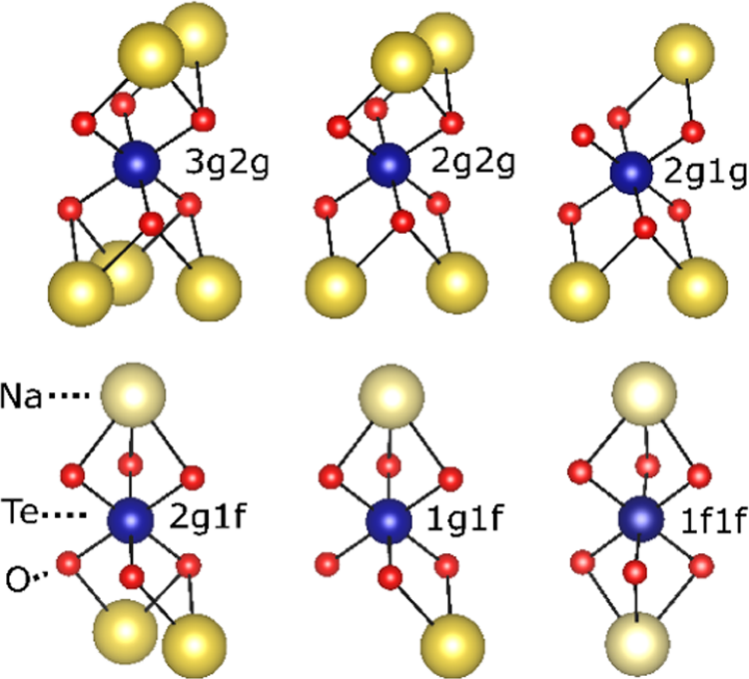
Five Te environments, in addition to the 1*f*1*f*-environment. As noted, the 3*g*2*g* is not common but is included to calculate the influence of the number of Na neighbors to Te. Note: there are 12 distinct Te configuration environments in total.

To progress further with analyses of the ^125^Te spectra, DFT calculations of chemical shifts were carried out as we expected ^125^Te chemical shifts to be influenced by the distribution of Na ions between the *g*-, *f*-, and *a*-sites due to its many electrons. Out of these sites, Na atoms in the *a*-sites have the largest separation from the Te site. Hence, for the NMR spectra, focus was on the *f*- and *g*-sites. We describe the Te environment with respect to interlayer Na cations with the notation *namb*, where *na* and *mb* refer to two neighboring sodium layers; *a*, *b* denotes the type of Na site (*f* or *g*) and *n*, *m* describes the number of Na in the said type of site (0, 1, 2, 3). In the case of an *f*-site, only *n*, *m* = 0, 1 are possible. When *n* = 0 or *m* = 0, either *na* or *mb* is omitted for simplicity. Some possible coordination environments for Te are shown in Figure 5.

Calculations to benchmark various approaches were performed on the most stable configuration reported in our previous work.^13^ This arrangement is a modification of the hexagonal structure, with twice as many atoms in the unit cell to account for a nonhexagonal reconstruction of the Na sublattice with Na ions equally distributed across the *g*- and *f*-sites and with *a*-sites unoccupied. In this model, all Te atoms exhibit a 2*g*1*f* coordination environment.

Obtained benchmarking chemical shift data are reported in Table 2 and compared with experimental values. The GGA calculation correctly reproduces the sign of the chemical shift and its order of magnitude but deviates clearly from the experimental reference. It is hence tested whether a different setup for the calculation would provide a chemical shift closer to the measured values.

**Table 2:** Calculated Shifts According to Three Methods for the 2*g*1*f* Environment in NZTO. TeO_2_ is Used as a Reference in the Computations, While Te(OH)_6_ is the Standard Used for Referring Te Shifts and in Experiments

method	shift from TeO_2_ [ppm]	shift from Te(OH)_6_ [ppm]
GGA (standard setting)	–291	460
GGA + core states	–278	473
SCAN + core states	–609	142
experimental span NZTO	–606 to −587	145–164

The most accurate set of PAW potential is adopted. In this approach, labeled as “GGA + core states” in Table 2, the most external full shells of p and s electrons are treated as valence electrons, and the associated Kohn–Sham equations are explicitly solved. This method did not provide a shift value closer to the experiment. We note that the usage of hybrid functional (i.e., approaches mixing PBE and Hartree–Fock results when computing the exchange portion of the energy) is not viable due to the size of the system (4 formula units, i.e., 44 atoms). A more suitable option is the Meta-GGA functionals using the Laplacian of the density or the kinetic energy on top of the density and its gradient considered by GGA. Within this class of functionals, SCAN recently emerged as an approach capable of an accurate description of bonding in various cases, including oxides such BaTiO_3_, PbTiO_3_, and BiFeO_3_,^50^ while maintaining a computational cost comparable to GGA. The reason for this improvement in accuracy is, however, not obvious. While SCAN usually provides more accurate interatomic distances than GGA, the computed values are, in both cases, compatible with experimental data, as shown in Table S5.

The SCAN-based description of the chemical bonding, Table S6, is compatible with the GGA reference and shows a substantial ionic character in good agreement with expectations. The calculated Te shift is fully compatible with experimental references. For this reason, we use the SCAN method and proceed to a modified Te environment to compute the effect on the chemical shift. These environments are constructed to generate different Te coordination environments and are explained in detail in Section S5.

**Figure 6:**
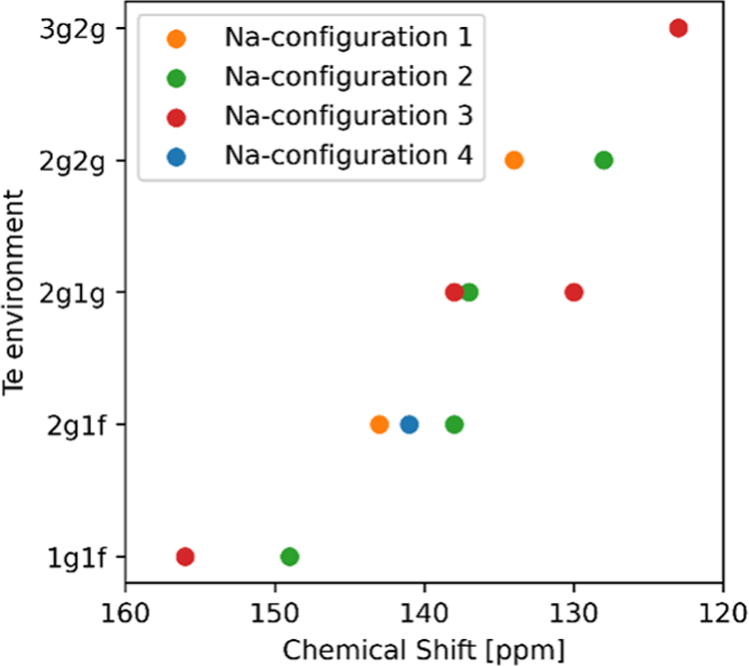
Calculated chemical shift values for five Te environments in NZTO that are produced by four different Na configurations as explained in more detail in Section S5.

Due to the small size of the system, only a few configurations can be explored. We have opted not to use a supercell system since a large number of atoms in a linear response calculation of this kind significantly increases the computational cost, and convergence of the self-consistent field computation is hard to achieve. The number of atoms is kept constant, and the Na distribution is rearranged to obtain the most frequently observed coordination environments for Te (computed by analysis of MD trajectories described in Section 3.5). These coordination environments are 1*g*1*f*, 2*g*1*f*, 2*g*1*g*, and 2*g*1*g*. The 3*g*2*g* environment, albeit rarely encountered, is also considered to be better, influencing the number of Na ions in the Te coordination environment. Figure 6 below shows the calculated chemical shift values for five different Te environments in NZTO.

There are two noticeable trends for the Te chemical shifts seen in Figure 6. Te atoms with a larger number of Na neighbors are more shielded and therefore shifted to a lower frequency compared to Te atoms with fewer Na neighbors. A comparison of the 2*g*1*f* and 2*g*1*g* environments is the only instance where the difference between the *f*- and *g*-sites can be determined, as one set of neighbors (2*g*) is the same for both, while the second is either *f* or *g*. In this situation, the edge-sharing *g*-position provides better shielding than the face-sharing *f*-site. Overall, the trend in chemical shifts can be summed up as follows:

1. Less shielding of Te is provided by fewer Na neighbors, which shifts the spectra to higher frequencies/ppm values.
2. Less shielding of Te is provided by Na atoms in the *f*-position (face-sharing) than those in the *g*-position (edge-sharing). This shifts the spectra to higher frequencies/ppm values.

On one side of a Te atom, there can be one, two, or three Na ions in *g*-sites or only one in an *f*-site. Hence, the 1st point is only relevant for environments involving *g*-sites.

We observe that for a given Te environment, the calculated shift is different depending on the Na configuration, i.e., it is influenced by Na positions beyond those that are directly face- or edge-sharing to the given Te atom. Since relatively small periodic systems are used for these computations, the Na distribution exerts certain distortions on the lattice, expectedly to be a feature of the simulations.

Based on these trends, we expect that Ga^3+^ substitution at the Zn^2+^ sites, which in turn will lower the amount of Na^+^, will result in a change in the peak intensities in the ^125^Te MAS NMR spectra toward higher frequencies, which is exactly what is observed in Figure 4. The main peak (Te with zero close Ga neighbors) shifts to the left with increasing substitution, consistent with a lower Na content.

Albeit we observe just a moderate shift of 2 ppm in the peak position, the width of the main peak broadens on Ga substitution. This may be due to increased dipolar couplings of Te with substituted Ga, as all Ga atoms have nuclei with magnetic moments, and only about 4% of Zn atoms have nuclear magnetic moments. The Te–Ga interactions, in addition to the already in place Te–Na interactions, and the relative strengths may be discussed by considering the magnetogyro ratios of ^125^Te, ^69,71^Ga, and ^23^Na (all are quite similar) and distances from Te to these elements (see details in SI9). The Te–Na and Te–Zn/Ga distances from the average structure from XRD are 2.81 and 3.05 Å, respectively. Hence, by increasing the Ga content, one could expect a relative increase in nuclear dipole–dipole couplings of Te, albeit complicated due to Ga’s relaxation rates. Furthermore, Na is known to be dynamic at room temperature in these materials, and Na’s interactions with Te are therefore decreased relative to a static case. The result is that when Ga is substituted into the material, one could expect a broadening of Te peaks due to the increase in dipole–dipole couplings of Te and nearby nuclei.

### 3.5 *Ab Initio* MD Simulations: Na Distribution and Te Environments

It is not possible to break down ^125^Te spectra into an unambiguous decomposition, and DFT simulations are performed to gain some insight into the Te coordination environments. Three compositions of Na_2–*x*_Zn_2–*x*_Ga*_x_*TeO_6_ type with *x* = 0 (NZTO), 0.083 (2Ga), and 0.167 (4Ga) were explored with *ab initio* MD simulation, the latter two with, respectively, 2 and 4 Ga^3+^ ions in a supercell with 24 formula units. The construction of the systems is explained in Section S7. For each composition *x*, we consider two simulation temperatures, 750 and 1000 K. As demonstrated in Section S7, the 4Ga system exhibits a clear preference for an uneven Na distribution when comparing Na layers. In this case, a 23:21 distribution of the 44 Na ions has an energy gain of 0.2 eV with respect to a system with an even (22:22) distribution. The layer with a larger vacancy concentration exhibits an increased occupancy of the *a*-site.

**Figure 7:**
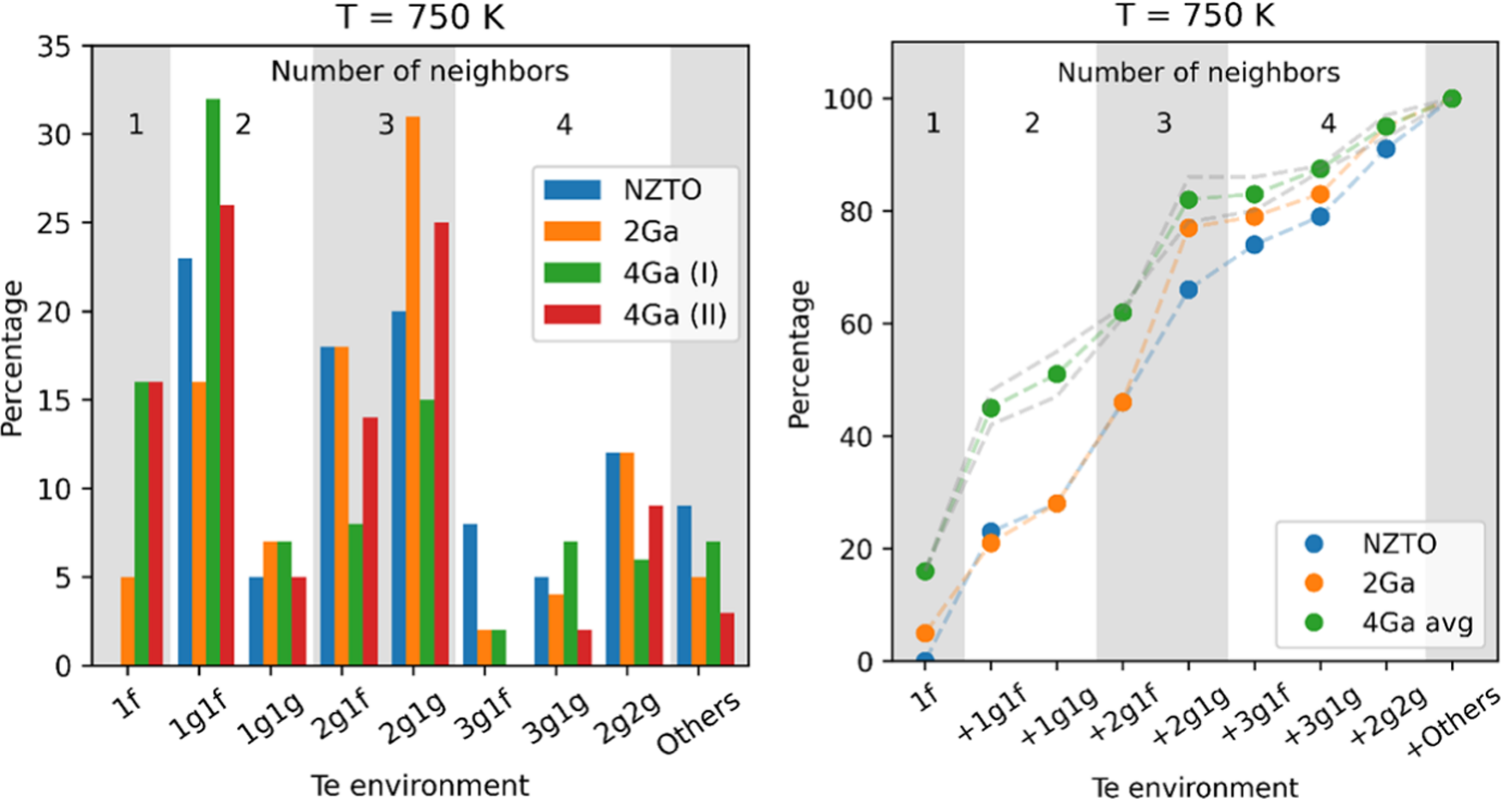
Te environments from 50 ps MD at 750 K. The number of Na neighbors around Te is marked as gray and white areas as a guide to the eye. (a) Relative percentages of each Te environment; (b) cumulative distribution, where amounts of each environment are added in the order of calculated shielding from Section 3.4. The average of two 4Ga runs is plotted, with the two individual runs indicated by gray dotted lines.

The average occupation of the Na sites is computed from the AIMD trajectories using an *ad hoc* procedure described in S8 and reported in Table S7. When examining these data, it is important to keep in mind the multiplicity of the three sites: if the occupancy of all of the available sites were equal, the sum of the *g-*sites would be three times as populous as that of the *a*-site. At 750 K, we note that an increased amount of Ga corresponds to a more favorable occupation of the *a*-sites. At 1000 K, the Na site population of all systems is compatible, and it is impossible to identify any effect of Ga substitution. Using the described labeling of the occupied Na sites, we can determine the coordination environments of Te from the AIMD trajectories and compute their abundance using an extension to the algorithm described in Section S8. The resulting population for 750 K is shown in Figure 7a. At this temperature, the Ga substitution influences the Na distribution.

For the pure NZTO, the more populous environments are 1*g*1*f*, 2*g*1*f*, 2*g*1*g*, and, to a minor extent, 2*g*2*g*, whereas the 1*f*1*f* is not populated at all. As the *f*-site has an expected high occupancy, this may then indicate that the Na layers are not independent but that the Na in one layer influences the structure in adjacent layers. In 4Ga, it is interesting to note that the 1*f* environment becomes very populated as the *f-* and *a*-sites become more favorable. As shown in Figure 7b, we further note that the 2*g*1*g* environment becomes highly populated for 2Ga.

With the limited size of the supercell, the amount of each environment is quite low, and the exact number is therefore quite uncertain. Instead, one can look at the general trends, which are clearer when looking at the cumulative distribution of Te environments in Figure 7b, plotted in the order of the shielding calculated in Section 3.4. This plot demonstrates that the samples with the high level of Ga substitution have more of the Te environments with a low number of Na neighbors, while the unsubstituted material has more of Te environments with a high number of Na neighbors. This is fully in line with the interpretation of the ^125^Te NMR data: the spectra will gain more intensity in the high-frequency region (left part) upon Ga substitution due to an increase of Te environments with fewer Na neighbors. It should be noted that this is not a peak shift but a relative change in the peak intensity ratios for different environments. This could be the explanation for the disappearance of the shoulder peak at 145 ppm, which is due to Te environments with a high number of Na neighbors for the materials with a lower amount of Na. This is also consistent with the observed features in the Te spectra, where the environments do not vary linearly with composition *x*. The peak maxima shift minimally, but the edges of the peak shift to a larger extent. The shift of the high-frequency edge is well in line with the increased population of the 1*f* environment, which is expected to be at a higher frequency than any environment in the pure NZTO. Furthermore, it is very likely that the ^125^Te NMR spectra consist of multiple overlapping peaks, which are not resolved.

### 3.6 Comments on Ion Conductivities Based on New Synthesis Protocol: Comparison and Discussion

The homogeneity of Ga in the Ga-substituted materials is likely not to have a large direct influence on the total ionic conductivity as measured by, e.g., impedance measurements. The synthesis method we have reported is employed to control the stoichiometry, ensuring that no region has a lower degree of substitution, which in turn might give a local decrease in ionic conductivity. The bulk (interlayer) conduction is based on the large number of vacancies relative to the Na number, which should be comparable if samples are stoichiometric. A comparison between the ^23^Na NMR for NZTO synthesized with sol–gel and solid-state synthesis is shown in Section S10, which demonstrates the same local environment. This suggests that the different synthesis methods have very little influence on the local structure, which would result in a relatively similar bulk conductivity. However, there are other influences on the total ion conductivity where the synthesis method is believed to have a greater influence. One is the grain boundary conductivity, which was demonstrated by Wu et al. to be 2 orders of magnitude lower than that of the bulk, greatly limiting the total conductivity.^15^ The temperature program used in this work has a shorter heating time than previous studies, which would yield smaller particles, resulting in more grain boundaries and thus might reduce total ionic conduction. Material preparation aiming for high ion conductivity might therefore have to be further optimized to give large particles and dense samples, which should be possible as the sol–gel synthesis method can tailor particle size and morphology.

## 4 Conclusions

In this work, we have described sol–gel synthesis and structural characterization of layered NZTO and its Ga-substituted derivatives (Na_2–*x*_Zn_2–*x*_Ga*_x_*TeO_6_, *x* = 0.00, 0.05, 0.10, 0.15, 0.20) by means of XRD, MAS NMR, and DFT methods.

The sol–gel synthesis protocol we applied provided samples with a high level of purity and with a homogeneous distribution of Ga. This is essential for the manufacturing and for the detailed characterization of a solid-state electrolyte candidate material, in particular when it comes to relationships between composition (substituents) and structure (sites).

The NMR data, with the support of DFT modeling, suggest a uniform Ga substitution; hence, we can claim that Ga atoms take the position of the Zn atoms with a concomitant reduction of the Na content. The introduction of heterovalent Ga atoms influences the Na substructure, in a way that the various Wyckoff sites become more energetically similar. The DFT modeling also suggests an inhomogeneous Na distribution between the neighboring layers for higher substitutions, in addition to an influence on the Na distribution in one layer from the Na structure in the adjacent layers.

The ^125^Te NMR chemical shift values are influenced by both Na and Ga. The effect created by the Na distributions in the two neighboring interlayers was analyzed by DFT calculations. We found that linear response calculation using the SCAN functional gave values for the chemical shifts within the experimental range. The Ga-containing samples showed additional peaks in the ^125^Te NMR spectra in quantities as expected for a uniform substitution mechanism.

## Supporting Information Available

Supporting Information with details on DTF modeling, Rietveld refinement, SEM images, curve fitting of ^23^Na and ^125^Te spectra, Te configuration for chemical shift calculations, discussion of GGA and SCAN functionals, starting points for DFT modeling of Ga-doped materials, dynamical definition of Na and Te coordination environments, notes on evaluation on dipolar couplings, and comparison between ^23^Na spectra of NZTO synthesized using sol–gel and solid-state synthesis (PDF)
