# Supplementary material for: Effects of Ga Substitution on the Local Structure of Na2Zn2TeO6
Source: Inorg Chem. 2022 Aug 9;61(33):13067–76. doi: 10.1021/acs.inorgchem.2c01431 (PMC9400102; doi:10.1021/acs.inorgchem.2c01431)
Supplement: Supplementary file 1 — ic2c01431_si_001.pdf [file ic2c01431_si_001.pdf]

Supplementary information:

Effects of Ga-substitution on the local structure of $Na_2Zn_2TeO_6$

Frida Sveen Hempel,[a, b] Federico Bianchini,[b,†] Bjørnar Arstad *[a] and Helmer Fjellvåg *[b]

a) SINTEF Industry, Forskningsveien 1, 0373 Oslo, Norway

b) Department of Chemistry and Center for Materials Science and Nanotechnology, University of Oslo, Oslo 0371, Norway

† Current address: Center for Bioinformatics, University of Oslo Gaustadalléen 30 N-0373 Oslo

* bjornar.arstad@sintef.no

* helmer.fjellvag@kjemi.uio.no

S1 DFT modelling

S1.1 Summarized information of calculations

The simulations were performed using VASP version 5.4.4,[1–4] with input/output manipulation and data analysis performed using python version 2.7.17, ase version 3.17.0 and quippy (https://github.com/libAtoms/QUIP.git ad62f721a-dirty) which was compiled on linux_x86_64_gfortran architecture.

Table S1.1: Input keywords for all calculations. Some additional information on the AIMD simulations: The thermalisation uses a *NVT* ensemble, with *T* specified in the INCAR. SIGMA of Fermi function is adjusted to the desired *T*. The MD calculation restarted until the desired thermalisation time is reached, from the CONTCAR to get the positions and momenta. The electronic problem is restarted from scratch every time. This saves time, as we are avoiding writing charge density and wave function, which is extremely time-consuming and disk-space consuming for a system of large size. The production uses a *NVE* ensemble, which is restarted from thermalized configuration. *T* is taken from the momenta of the thermalized system, but the SIGMA of the Fermi function is kept from the *NVT* run, as not to modify the electronic problem. This is restarted until desired time length is reached.

SCAN		Structural optimization	AIMD	
Structural optimization	Chemical shift		Thermalization	Production
KSPACING = 0.4	PREC = A	ENCUT = 500.000000	ENCUT = 400.000000	ENCUT = 400.000000
PREC = A	IALGO = 38	KSPACING = 0.400000	EDIFF = 1.00e-07	EDIFF = 1.00e-07
IBRION = 2	IBRION = -1	SIGMA = 0.100000	PREC = Normal	PREC = Normal
ISIF = 3	ISIF = 2	EDIFF = 1.00e-04	GGA = PE	GGA = PE
ISTART = 0	ISTART = 0	ALGO = Very_Fast	ALGO = Very_Fast	ALGO = Very_Fast
ENCUT = 600.0	LCHARG = .FALSE.	GGA = PE	ISYM = 0	ISYM = 0
EDIFF = 1E-5	LWAVE = .FALSE.	PREC = Normal	NCORE = 10	NCORE = 10

ISMEAR = 0	LCHIMAG = .TRUE	IBRION = 2	ISTART = 0	ISTART = 0
SIGMA= 0.1	ENCUT = 600.0	ISIF = 2	NSW = 5000	NSW = 5000
LREAL = A	EDIFF = 1E-8	ISMEAR = 1	IBRION = 0	IBRION = 0
NSW=40	ISMEAR = 0; SIGMA= 0.1	ISTART = 0	ISIF = 2	ISIF = 2
METAGGA = SCAN	PREC = A	NSW = 300	LCHARG = .FALSE.	LCHARG = .FALSE.
IALGO = 38	DQ = 0.001	NCORE = 8	LWAVE = .FALSE.	LWAVE = .FALSE.
LASPH = .TRUE.	ICHIBARE = 1	LCHARG = .FALSE.	LREAL = Auto	LREAL = Auto
LDIAG = .FALSE.	LNMR_SYM_RED = .TRUE.	LWAVE = .FALSE.	ISMEAR = -1	ISMEAR = -1
NELM=120	NSLPLINE = .TRUE.	LREAL = Auto	SIGMA = 0.086	SIGMA = 0.086
NELMDL = 10	LREAL = A		POTIM=1	POTIM=1
	KSPACING = 0.4		SMASS=-1	SMASS=-3
	METAGGA = SCAN		TEBEG = 1000	NBLOCK = 1
	LASPH = .TRUE.		TEEND = 1000	
	LDIAG = .FALSE.		NBLOCK = 50	
	NELM=200			
	NELMDL = 10			
	AMIX = 0.1			
	BMIX=0.0001			
	IMIX = 1			
	ISYM=0			

All files are available upon request.

S1.2 Parameters for linear response calculations (chemical shift)

The following electron configurations are considered as valence: $2s^2 2p^6 3s^1$ for Na, $3s^2 3p^6 3d^{10} 4s^2$ for Zn, $4s^2 4p^6 4d^{10} 5s^2 5p^4$ for Te, and $1s^2 2s^2 2p^4$ for O. Integration over the Brillouin zone (BZ) is performed using Γ-centred Monkhorst-Pack grids[5] with a resolution of 0.4 $Å^{-1}$. Electronic gaussian smearing[6] with a 0.1 eV broadening width is used. A 600 eV kinetic energy cut-off for the plane wave expansion is chosen. A thick grid for Fast Fourier Transform is used, with twice as many points as the ones required by the cut-off energy. The blocked-Davidson scheme is used for optimising the orbitals and real space projection operators are used to evaluate the non-local part of the pseudopotential. The systems were optimised with an energy convergence threshold of 10^{-4} eV, while the energy convergence threshold of the self-consistent field (scf) calculation is set to 10^{-5} eV. The accuracy of scf calculation is further increased for the linear response calculations, with an energy convergence threshold of 10^{-8} eV.

S1.3 Parameters for structural optimisation

The following electron configurations are considered as valence: $3s^1$ for Na, $3d^{10} 4s^2$ for Zn, $4s^2 4p^1$ for Ga, $5s^2 5p^4$ for Te, and $2s^2 2p^4$ for O. Integration over the Brillouin zone (BZ) is performed using Γ-centred Monkhorst-Pack grids[5] with a resolution of 0.4 $Å^{-1}$. Electronic smearing is introduced using the Methfessel and Paxton method with a 0.1 eV broadening width. A 500 eV kinetic energy cut-off for the plane wave expansion is chosen. The orbitals are optimised using the Residual minimization method direct inversion in the iterative subspace[7] and real space projection operators are used to evaluate the non-local part

of the pseudopotential. The systems were optimised with an energy convergence threshold of 10^{-3} eV, while the energy convergence threshold of the scf calculation is set to 10−4 eV. The volume of the cell is kept fixed upon optimisation.

S1.3 parameters for AIMD simulations

The parameters form 6.1.2 are re-used here with some adjustments: the energy cut-off is decreased from 500 to 400 eV given the computational cost of these calculations. Moreover, the Brillouin Zone is sampled only using the Gamma point. We are consequently using the VASP version with real (not complex) numbers, corresponding to a better computational efficiency for this type of system. The convergence threshold of the scf loop is set to 10^{-7} eV. While this increases the computational cost, it prevents a drifting of the total energy that was observed upon testing. The dielectric function, used as a charge density mixer, is not reset after each ionic update to reduce the number of self-consistent steps after the first ionic interactions. Finally, the electronic smearing is introduced via the Fermi-Dirac distribution matching the electronic temperatures to the desired ionic temperatures.

AIMD simulations are executed for the starting system and two replicas of it, in which the volume has been expanded by 1% and 2% without modifying the shape of the cell (i.e. applying a diagonal strain). The dynamics is then propagated for 5~ps to allow for a (partial) thermalisation. After this step, the dynamics is further propagated for 5 ps more, without changing any parameters and collecting the values of the stress tensor at each step. These are averaged and used to estimate the equilibrium cell shape at a given temperature through linear fitting. This computation of the anisotropic thermal expansion is not accurate, but allows to define a reliable starting system at a reasonable computational cost without modifying the input parameters of the simulation: obtaining a more accurate estimate, e.g. through phonons, would require an entirely difficult setup of the simulation. Moreover, the occupation of the Na sites changes with time, and it is thus advisable to work on system averages rather than considering a structural minimum. A fourth replica of the initial system is then generated using the target lattice parameters. The stress on the cell is verified for this system using the same method. It is found that diagonal stress values are always smaller than 10 GPa and the average values are close to zero. The diagonal elements of the strain tensor are always negligible, being rarely larger than 1 GPa for all the fourth replicas. The simulation for the final replica is then protracted for 10 ps more, before switching to the microcanonical ensemble. The first 10 ps of dynamics in this ensemble are also considered part of the thermalisation stage and not accounted for when computing the physical quantities of interest e.g. the mean square displacement.

S2 Rietveld refinement

Table S1: Measurement and Rietveld refinement, where shared values are given in shared values. Peak

	x				
	0.00	0.05	0.10	0.15	0.20
Chemical formula	$Na_2Zn_2TeO_6$	$Na_{1.95}Zn_{1.95}Ga_{0.05}TeO_6$	$Na_{1.9}Zn_{1.9}Ga_{0.1}TeO_6$	$Na_{1.85}Zn_{1.85}Ga_{0.1}TeO_6$	$Na_{1.8}Zn_{1.8}Ga_{0.2}TeO_6$
Formula weight	400,33	399,40	398,47	397,54	396.6
a, b	5.29034(4)	5.28165(3)	5.27215(5)	5.27085(4)	5.26372(8)
c	11.24233(15)	11.24670(13)	11.25116(19)	11.24508(15)	11.2591(3)
α, β			90		
γ			120		
Source			laboratory X-ray		
Temperature			22°C		
Wavelength			1,540598 Å		
Crystal system			Hexagonal		
Space group			no. 182, *P6₃22*		
Z			2		
d-space range			0.85 - 8.9		

Table S2: Fit values for inclusion of anisotropic size broadening and preferred orientation, which provides at best marginal improvement, and sometimes increase of R_p values. The particles are clearly anisotropic, as seen in figure S2, and while the size of the crystallites is unknown it is natural to assume some anisotropic size broadening, as described by Katerinopoulou et al.[8] Including this in the model does not improve the fit significantly. Preferred orientation seems to be sufficiently reduced in the capillary geometry. None is therefore included in the further refinement.

Parameter	Model	*x*				
		0.00	0.05	0.10	0.15	0.20
	Normal	18,25	11,08	13,70	11,14	20,54
	Anisotropic size	17,68	11,16	15,43	11,03	20,33
R_p	Preferred orientation	17,51	10,56	17,66	10,69	18,68
	Normal	23,28	14,29	17,66	14,49	25,76
	Anisotropic size	22,88	14,47	19,77	14,57	25,70
R_{wp}	Preferred orientation	22,37	13,65	17,07	13,97	24,01
	Normal	2,45	2,30	1,99	1,92	2,17
	Anisotropic size	2,36	2,36	2,50	1,94	2,16
χ^2	Preferred orientation	2,26	2,10	2,36	1,78	1,88

Definitions of R-factors from Rietveld refinement. R-pattern R_p defined as

$$R_p = \frac{\sum |y_{O,i} - y_{C,i}|}{\sum y_{O,i}}$$

with $y_{o,i}$ and $y_{c,i}$ is the observed and calculated data at data point i. The R-weighted expected pattern, R_{wp} is defined as

$$R_{wp} = \sqrt{\frac{\sum w_i (y_{O,i} - y_{C,i})^2}{\sum w_i {y_{O,i}}^2}} \qquad w_i = \frac{1}{\sigma(Y_{o,\,i})^2}$$

where w_i is the weighing given to data point *i*, and the σ $(Y_{o,i})^2$ is the error is the error in $Y_{o,m}$. The R-expected, R_{exp}, is defined as

$$R_{exp} = \sqrt{\frac{M - P}{\sum w_i {y_{O,i}}^2}}$$

where M is the number of data point and P is the number of parameters. From this, the χ^2 is defined as

$$\chi^2 = \left(\frac{R_{wp}}{R_{exp}}\right)^2$$

This is often redefined to goodness of fit, G, which is defined as $G^2 = \chi^2$.

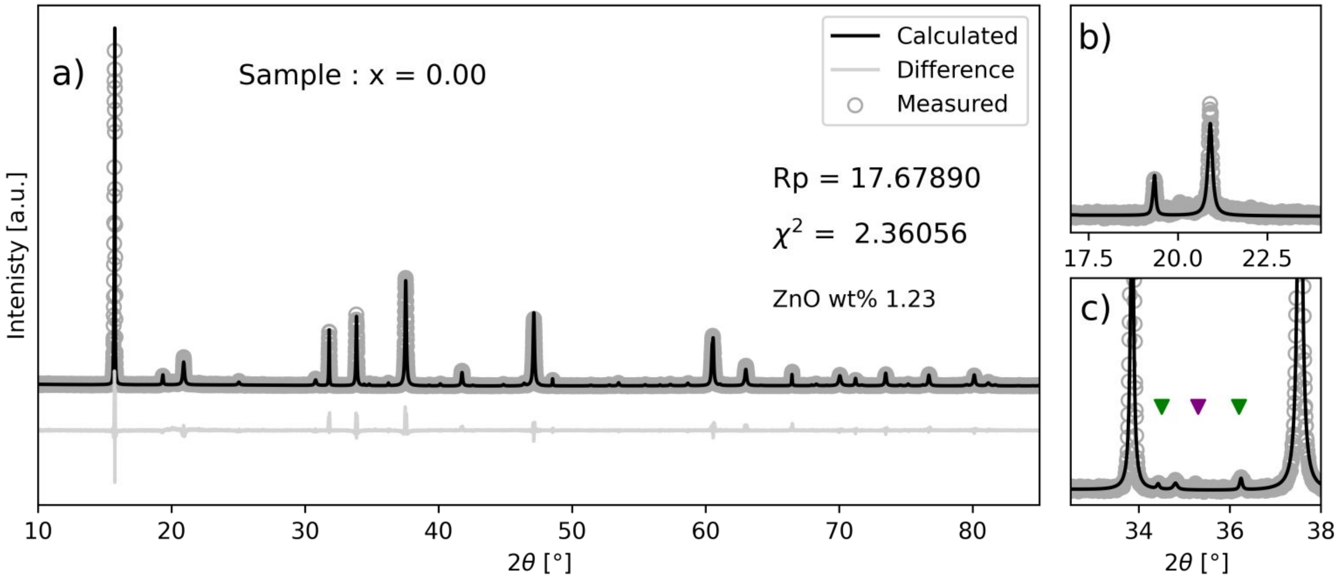
a)
Sample : x = 0.00
Calculated
Difference
Measured
Rp = 17.67890
χ^2 = 2.36056
ZnO wt% 1.23
Intenisty [a.u.]
2θ [°]
b)
c)

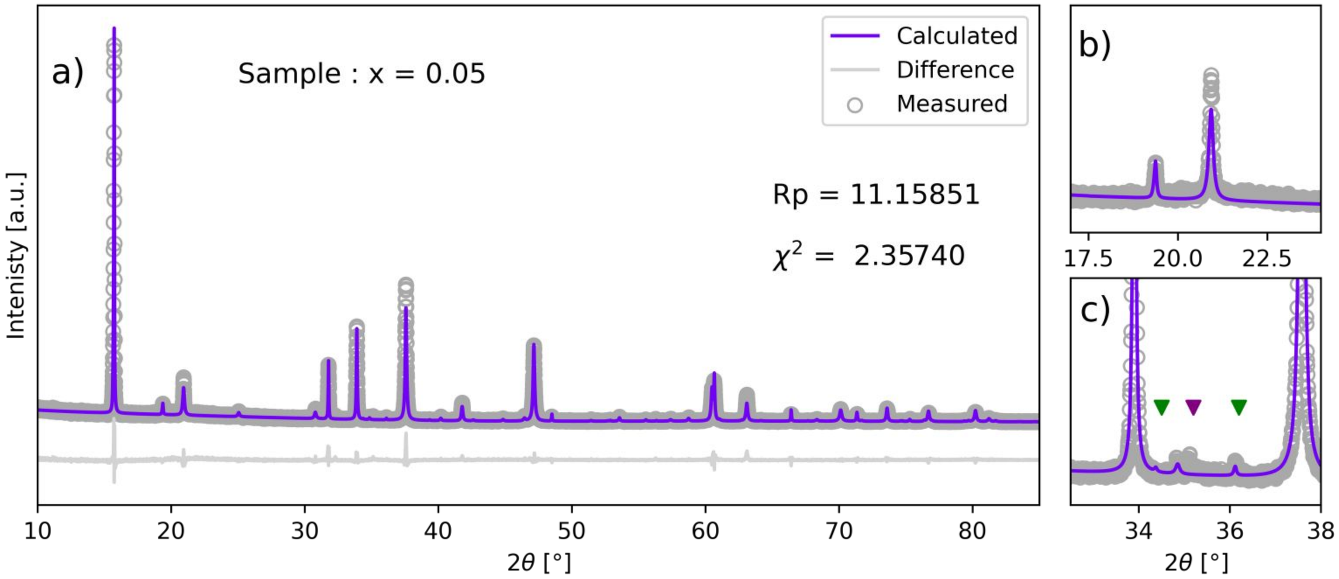
a)
Sample : x = 0.05
Calculated
Difference
Measured
Rp = 11.15851
χ^2 = 2.35740
Intenisty [a.u.]
2θ [°]
b)
c)

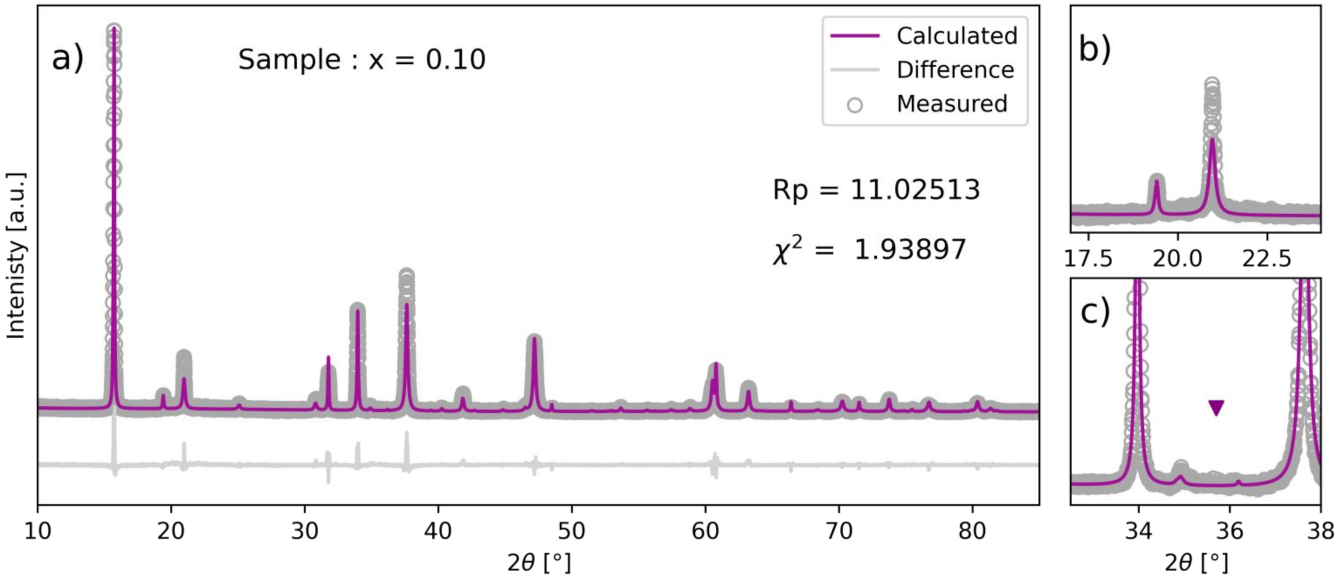
a)
Sample : x = 0.10
Calculated
Difference
Measured
Rp = 11.02513
χ^2 = 1.93897
Intenisty [a.u.]
2θ [°]
b)
c)

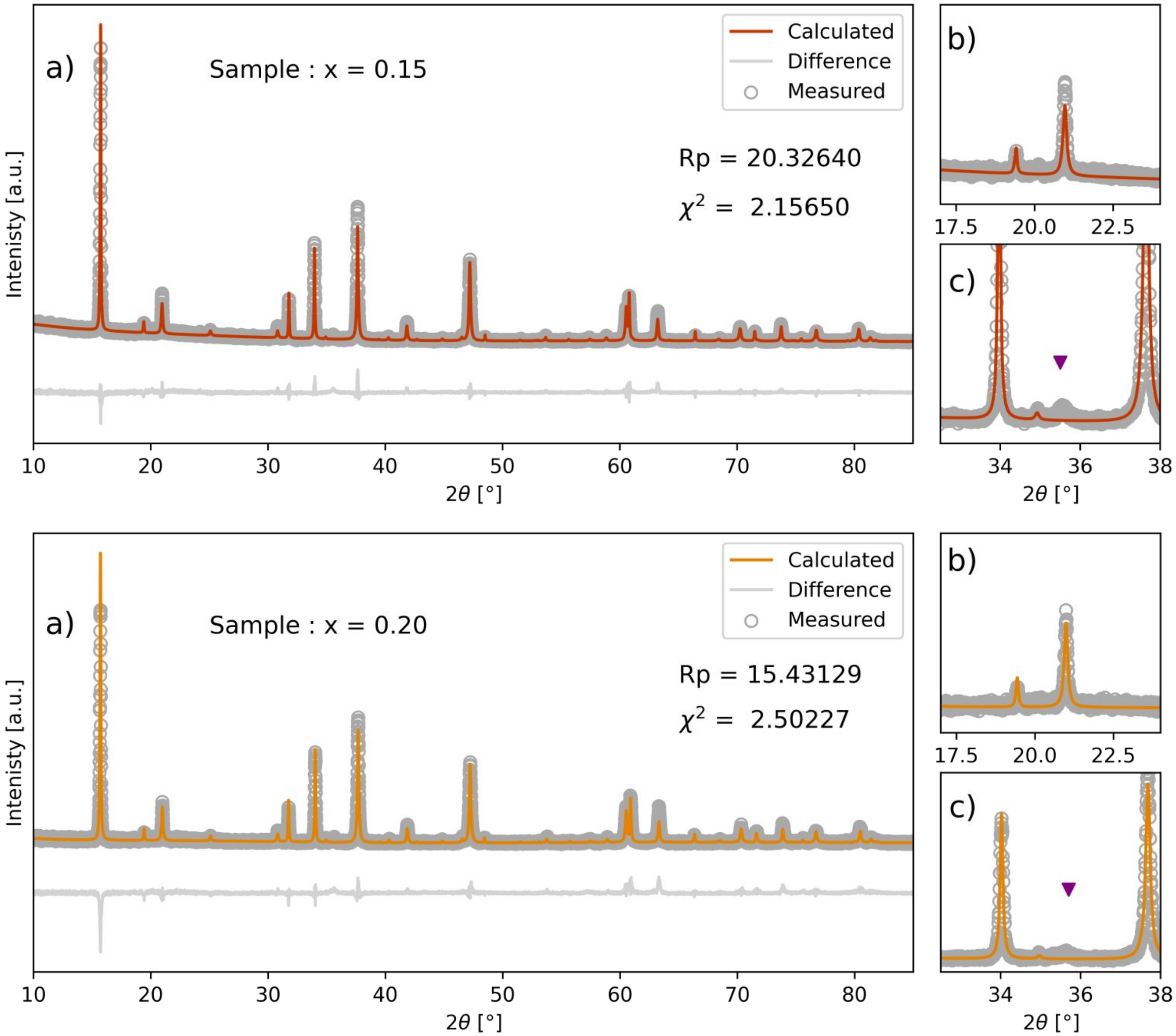

Figure S1: a) Conventional Rietveld refinement of all samples against XRD with the difference in grey. b) Inset of elevated background and extra reflections discussed in section 3.1., marked with blue arrows. c) Reflections from ZnO impurity, marked with green arrows, and unknown peak marked with purple arrow. Phase fractions are calculated by weight percentage, and calculation is not reliable below 3%. Only sample *x* = 0.00 has clear peaks related to ZnO. Sample *x* = 0.05 has indication of a peak, but the phase fraction is very low. The unknown peak marked with purple could be due to the Na-displacements previously reported

Table S3: Atomic coordinates and occupancies used in Rietveld refinement. Values adopted from Evstigneeva et al.[9] and not fitted, due to the low scattering power of Na.

Atom	Wyckoff symbol	Coordinates x	y	z	Occupancy	B_{iso}
Na1	6g	0.6615	0	0	0.28	1
Na2	2a	0	0	0	0.043	1
Na3	4f	1/3	2/3	0.5111	0.535	1
Te	2c	1/3	2/3	0.25	1	0.5
Zn1	2b	0	0	0.25	1	0.5
Zn2	2d	1/3	2/3	0.75	1	0.5
O	12i	0.3594	0.3312	0.6501	1	1

Table S4: Bond lengths calculated from DFT compared to experimental values from Rietveld refinement, for NZTO and 4Ga with x = 0.00 and x = 0.20 respectively. In both cases, Na-, Zn- and Te-O bonds decrease upon Ga-insertion, while the octahedral O-O around Zn. The DFT show that the prismatic O-O distance increase, which is the opposite of the decrease in the average structure.

	NZTO	4Ga	x = 0.00	x = 0.20
Na-O, g sites, layer 1	2.48 ± 0.11	2.48 ± 0.14	2.44	2.44
Na-O, f sites, layer 1	2.51 ± 0.18	2.49 ± 0.12	2.43	2.43
Na-O, a sites, layer 1	-	2.53 ± 0.11	2.49	2.49
Na-O, g sites, layer 2	2.48 ± 0.11	2.48 ± 0.18	-	-
Na-O, f sites, layer 2	2.51 ± 0.18	2.49 ± 0.13	-	-
Na-O, a sites, layer 2	-	2.61 ± 0.36	-	-
Zn-O distance	2.16 ± 0.04	2.17 ± 0.08	2.15	2.14
Te-O distance	1.97 ± 0.01	1.97 ± 0.02	1.97	1.97
Na-Zn, f sites, layer 1	2.88 ± 0.01	2.89 ± 0.02	2.69	2.69
Na-Te, f sites, layer 1	3.07 ± 0.01	3.06 ± 0.01	2.94	2.94
Na-Zn, a sites, layer 1	-	3.06 ± 0.09	2.81	2.81
Na-Zn, a sites, layer 2	-	3.1 ± 0.02		
O-O, prism, layer 1	3.47 ± 0.12	3.51 ± 0.08	3.38	3.28
O-O, prism, layer 2	3.48 ± 0.11	3.5 ± 0.08		
O-O, Zn octahedra	2.8 ± 0.11	2.88 ± 0.14	2.9	2.98
O-O, Te octahedra	2.74 ± 0.02	2.73 ± 0.04	2.77	2.86

S3 SEM

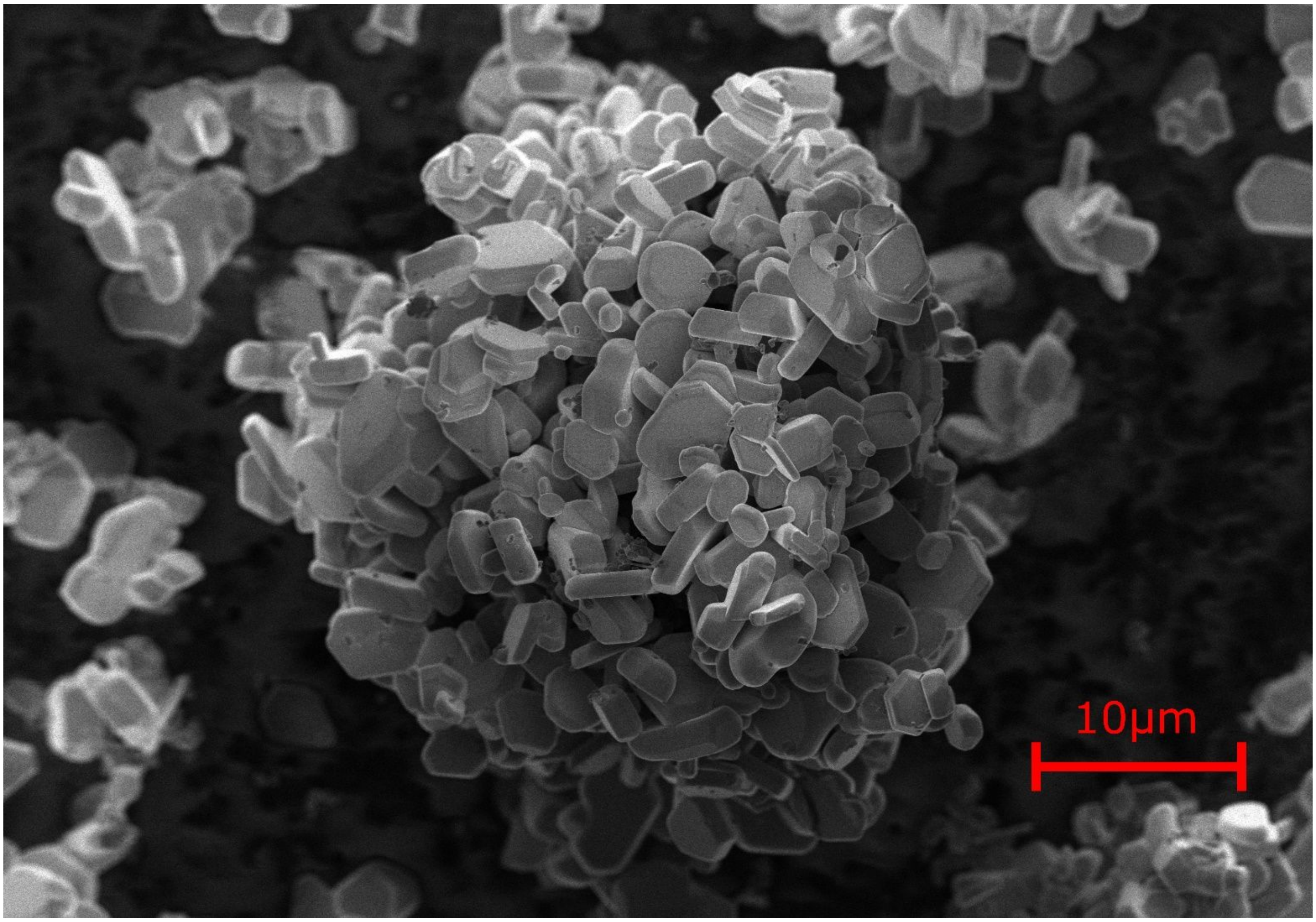

Figure S2: SEM image of NZTO.

S3 ^{23}Na NMR spectra breakdown from curvefitting

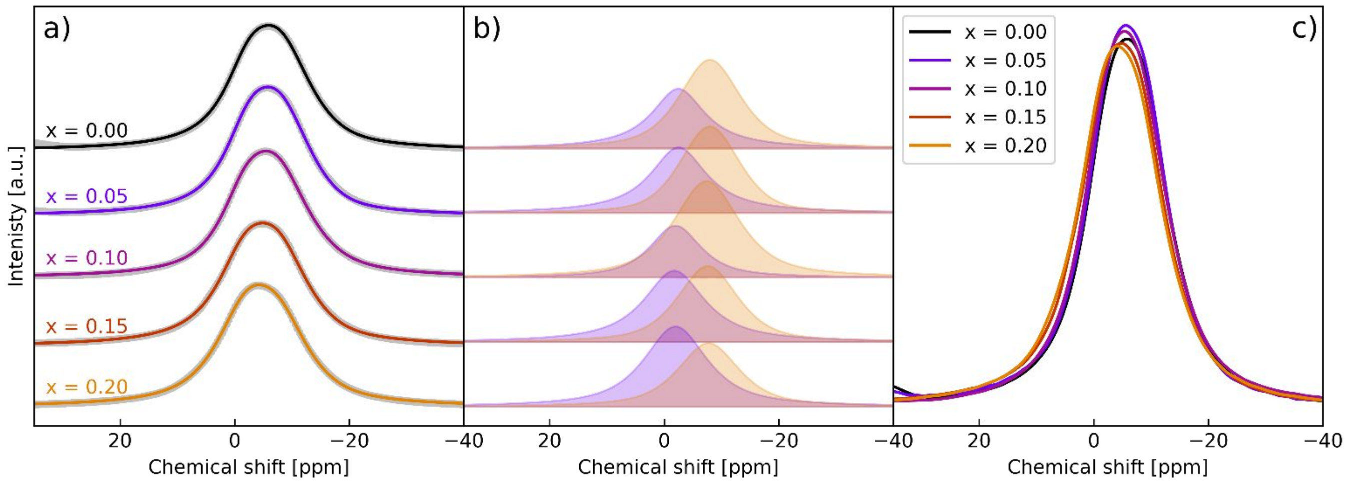

Figure S3: a) Fit of ^{23}Na NMR with grey points for spectrum and coloured line for fit. b) breakdown of ^{23}Na NMR spectra. The peak breakdown has multiple solutions which gives a similar quality fit. The observed peaks are not possible to fit with a single Gaussian peak, which means that if the lack of peak shape is due to a dynamic phenomenon, the peaks have not yet fully coalesced. c) Normalized ^{23}Na NMR spectra with no offset. The higher frequency side of the peaks shift to higher values with increasing substitution.

S4 ^{125}Te NMR spectra breakdown from curvefitting

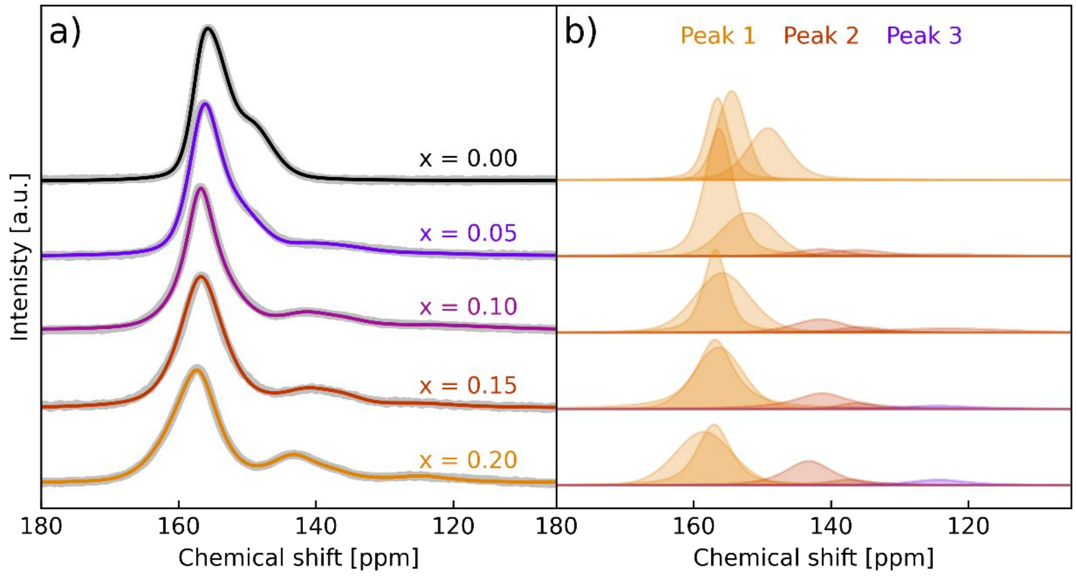

Figure S4: a) Fit and b) breakdown of ^{125}Te NMR. The breakdown is colour coded into the three groups, with the integrated relative frequency reported in Table 1. The peak breakdown has multiple solutions which gives a similar quality fit. The groups are, as explained in section 3.3, interpreted to be due to adjacent Ga-neighbours, but the peak breakdown does not have any physical interpretation and are only selected to fully describe peak shapes.

S5 Te configurations for chemical shift calculations

The first configuration is obtained by rearranging all the Na in one layer according to a honeycomb structure of *g*-sites, while the second layer maintains the non-hexagonal reconstruction. This configuration produces 2*g*2*g* Te coordination environments on top of the previously observed 2*g*1*f*. The second configuration is constructed by arranging the Na in one layer with a 3*g*1*a* pattern, where the three g sites are nearest neighbours and exhibit a triangular pattern. As in the previous case, the second layer maintains the original 2*g*2*f* pattern. This structure produces 4 distinct Te coordination environments: 1*g*1*f* , 2*g*1*g* , 2*g*2*g*, and 2*g*1*f*. In the last case, both Na layers have 3 *g*-sites occupied forming a triangular pattern. The remaining Na are placed in *a* and *f*, thus producing the following Te coordination environments: 1*g*1*f*, 2*g*1*g*, and 3*g*2*g*. After system relaxation, all these configurations have total energies that differ 24 meV or less with respect to the reference, meaning that they are meaningful local minima that can potentially be observed at room temperature. The last system included is the configuration used in the benchmarking.

S6 Comparison between GGA and SCAN results

Some properties of the NZTO systems are reported here to offer a comparison between the GGA and the SCAN functional. In table S3 the lattice parameters and the average Te-O bond length are reported. As expected, GGA overestimates the experimental values. SCAN, on the other hand, is shown to underestimate the values in this case, and it is not evident from these numbers which of the two is the best functional to describe the system. We further report in table S4 the charge population analysis as computed by the Bader method.[10] Also in this case, the results from SCAN and GGA are very similar and they are both very far from the ideal ionic picture. We conclude that GGA and SCAN are equally accurate in describing the basic properties of this system and the superior accuracy of SCAN is made evident only in the more demanding case of the linear response calculation for obtaining the chemical shift.

Table S5: Lattice parameters and interatomic distances as calculated by SCAN and GGA.

axis	SCAN	GGA	Experimental
a	5.25	5.33	5.29
b	9.07	9.21	9.16
c	11.14	11.34	11.24
Average Te-O bond length	1.939 pm 0.008	1.967 pm 0.008	1.971

Table S6: Bader charges computed using SCAN and GGA. The expected values from a purely ionic picture are reported for comparison

Ion	SCAN	GGA	Expected
Na	+0.9	+0.9	+1
Zn	+1.3	+1.4	+2
Te	+3.1	+3.3	+6
O	-1.2	-1.3	-2

S7 Starting points for configurations for Ga-doped NZTO systems

To create the starting point for the Ga-doped systems, each defect, impurity or vacancy, is introduced one after the other in the initial NZTO configuration: a 3x2x1 supercell system contains two Na- and two ZnTe layers. The Na is distributed according to the non-hexagonal pattern described in our previous work.[11] As a consequence of this, the Zn sites are not equivalent and more than one site needs to be considered for substitution with Ga. Two Zn sites are thus considered for the first Ga impurity: one between two unoccupied a sites and one between 2 f sites, one of which is occupied. Other configurations were not considered because (i) occupations for a sites is not so high in pristine NZTO, (ii) the occupation of both the f sites above and below never occurs (as demonstrated in section 3.5) and (iii) two unoccupied f sites sharing the same (x, y) coordinates are also rarely observed. The configuration with impurity between the two a sites is found to be more favourable by a small margin (10 meV). Both systems are thus considered further for generating the starting minima for AIMD calculations. The following step is the introduction of the first Na-vacancy. Since all the Na sites are in principle non-equivalent due to the presence of the impurity, all the possible sites in the structure have been considered and all the structures independently optimised. The optimisation procedure causes symmetry break in the Na sublattice not only in the layer containing the vacancy, but also in the other one. In both cases, the number of occupied g sites increases. This is a behaviour consistent with the g site being energetically more favourable [11] and with experimental evidence [12].

When the vacancy is introduced, the system with Ga over an *a*-site becomes more favourable by 50 meV, while maintaining a lower occupation of the *g*-site. The most favourable configuration is chosen in each case for further insertion of point defects.

The 2nd Ga-dopant is placed at all possible Zn-sites, as they are no longer inequivalent after introduction of the 1st. The system shows a clear preference for the systems with Ga in the previously undoped ZnTe layer, hinting that Ga is expected to be uniformly distributed in the structure. However, this is not conclusive, since our systems do not contain enough layers to further validate this point and, indeed, the integration of the peaks from ^{125}Te-NMR suggest inhomogeneous distribution for the x = 0.2. The most favourable configuration has both Ga impurities above *a*-site type, providing further evidence for the favourability of this site. Notably, also the system with impurity above the *f*-site has a second impurity above the *a*-site. While the two Ga-placements are comparable in energy, both systems show a preference for the placement of the next Na-vacancy in the same Na-layer as the 1st. However, the best 1:1 vacancy distribution is only a few meV above. For MD simulations, the Ga above *a*, 1:1 vacancy distribution will be used as it ensures better statistics (being the two layers equivalent) and is only

marginally less favourable. This is the system labelled 2Ga in the main text.

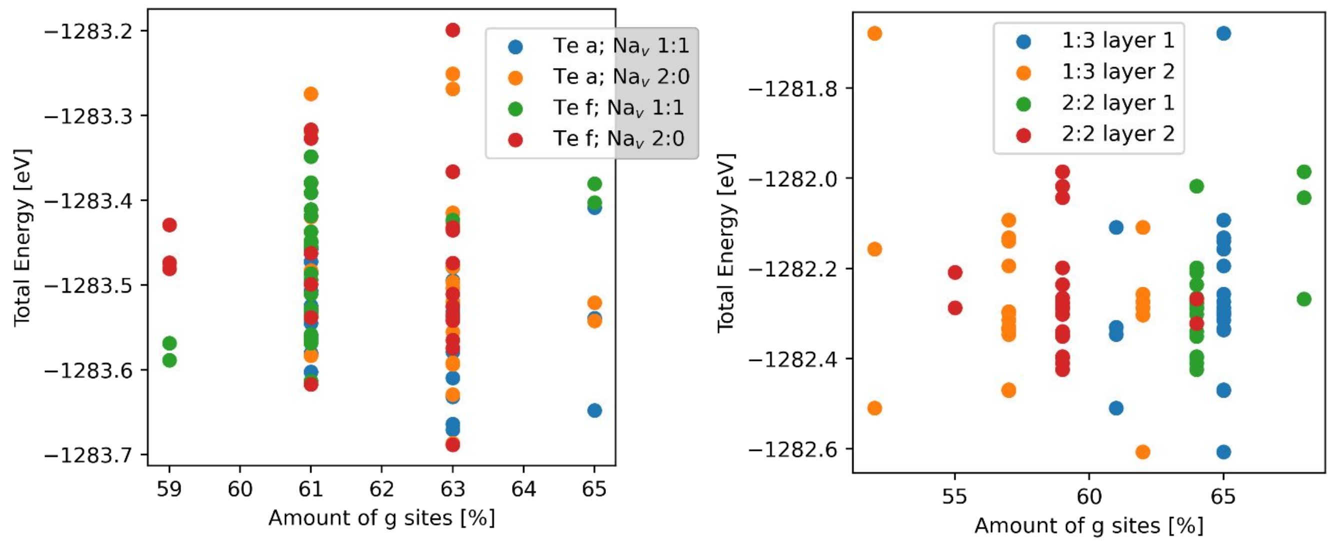

Figure S5: Na-distributions for the 2Ga systems (left) and the 4Ga system (right).

The 3rd most favourable Ga impurity is found to be placed between two *f*-sites where one is filled. The following Na vacancy is placed in the previously undoped layer, giving a 2:1 or 1:2 distribution. The last Ga-impurity exhibits the homogenous distribution, giving a 2:2 distribution between layers. The equilibrium in-layer Ga-distances are 8.16 and 5.3 Å, and there are no configurations with one NaO_6-prism sharing faces with two Ga-octahedra. The last Na vacancy shows a clear preference for unequal distribution, where the energy difference between the most stable 3:1 and 2:2 is quite large (0.2 eV). This 3:1 system, labelled 4Ga, is the one used as starting configuration AIMD simulations. Due to the unequal Na-distribution, the layers might behave differently. In order to obtain better statistics of the behavior of Na as a function of the Na density in the layer, we have decided to run 2 distinct AIMD simulations for this system and average over equivalent layers instead of considering system averages.

S8 Dynamical definition of Na sites and Te coordination environments

For each MD trajectory, we have considered a subset of 1000 configurations separated by 50 fs of dynamics. In each configuration, we define Na sites by averaging the (*x*, *y*) coordinates of atoms sharing the same *z*-coordinate, in particular (i) two Zn for the *a*-site, a Zn and a Te for the *f* site and two O for the *g* sites. This allows us to keep track of the ideal coordinate of the sites when the system is in temperature.

We then measure the distance between the actual positions of the Na during the dynamics and these ideal sites and project it in the (x, y) plane. The Na atoms are labelled *g*, *f* or *a* according to the shortest of these distances. This allows to compute dynamically the amount of Na at each site. The occupation of Na sites is averaged over the 1000 structures considered.

Having the Na atoms labelled by site in the AIMD trajectory, it is now possible to determine the coordination environment of Te and compute their abundance. As in the previous case, we use a subset of 1000 configurations to compute averages. For each Te atom, we have computed the modulus of the planar projection of the distance vector and selected the Na atoms within a 2.3 Å cut-off length. This is in line with the explanation of Te-environments explained in the introduction, where the *a*-sites do not contribute as they are not close enough to the Te atom. The same applies to the second shell of *f*-sites, which are as distant from Te as the *a*-site. The environments are then defined only by the amount of *g*- and *f*-sites, which cannot be simultaneously occupied in the same layer due to the proximity of these sites (≈ 1.7 Å).

Table S7: Na population computed from AIMD trajectories in the considered systems at selected temperatures. The multiplicity of each site is given, as the population will scaled both by the fractional occupancy of each site, as shown in figure 6, and the multiplicity of each site.

	Population [%] T = 750			Population [%] T = 1000		
System	3*g*	2*f*	1*a*	3*g*	2*f*	1*a*
NZTO	62	28	9	58	28	14
2GA (1vNa)	65	22	13	56	28	16
4GA (1vNa)	57	29	14	59	25	16
4GA (3vNa)	48	34	18	58	27	14

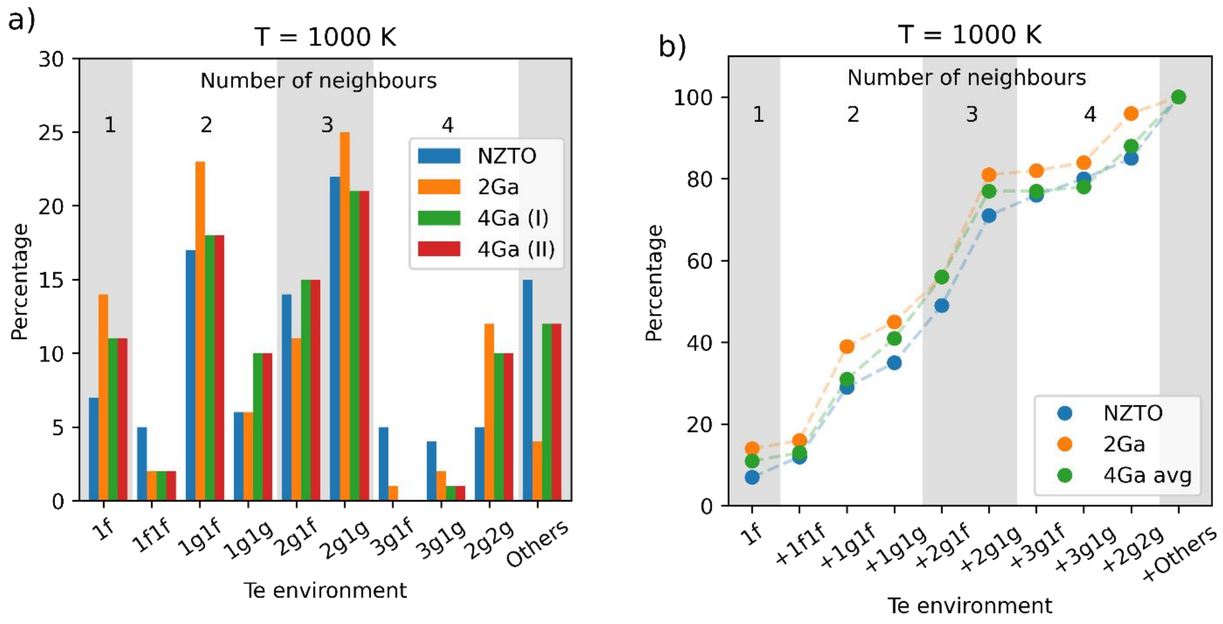

Figure S6: Te-environments from 50ps MD at 1000K. The number of neighbours is marked by grey and white area as a guide to the eye. (a) Relative percentages of each Te-environments, (b) The cumulative distribution, where the amounts of each environments is added together in the order of calculated shielding from section 3.4, which is done to highlight the changes between environments across samples, and how it relates to the Na-content. The two 4Ga runs is the same at 1000K as seen in a), and is therefore plotted as one in b).

S9 Dipolar coupling calculations

Below are average estimated bond lengths in Å extracted from average structures from Rietveld refinement of XRD:

Zn(Ga)-Zn(Ga)	Zn(Ga)-Te	Zn(Ga)-O	Zn(Ga)-Na	Te-Te	Te-O	Te-Na	O-O	O-Na	Na-Na
3.05	**3.05**	2.14	2.60	5.29	1.97	**2.81**	2.76	2.38	3.05

Out of these only those marked in bold should contribute most to interactions between magnetic dipoles of Te and neighbors, in this case Na and Zn/Ga. Both Zn and O are of low natural abundance regarding nuclei with magnetic moments, so we disregard these in this estimate.

From Bruker Table of Isotopes the gyromagnetic ratio, *γ*, for the most relevant nuclei are:

Isotope	Gyromagnetic ratio, γ [10^7 rad s^{-1} T^{-1}]	Natural abundance [%]
^{125}Te	-8.51	7.07
^{69}Ga	6.43	60.11
^{71}Ga	8.18	39.89
^{23}Na	7.08	100

The through space dipolar coupling constant is in angular frequency units:

$$D = \frac{\mu_0}{4\pi}\frac{h}{2\pi}\frac{\gamma_1\gamma_2}{r^3}$$

h is Planck's constant and μ_0 is the vacuum permeability. The interaction is proportional to the product of the magnetogyro ratios of the interacting nuclei and the cube of the inverse of distance.

$\gamma_1\gamma_2$ for Te*Ga or Te*Na are approximate ~ -55 (Te*^{69}Ga), ~ -70 (Te*^{71}Ga), ~-60 for Te*Na. The third power of the distances are respectively 3.08^3= 28, and $2,81^3$ = 22. As can be seen coupling-values are quite similar hence the average distance will be the major controlling factors. The Te-Te interaction has a denominator of about 5.29^3 = 148 and is therefore of significant lower strength.

From these estimates, for otherwise static atoms, the strongest single contribution for dipole-dipole through space interactions of the Te nucleus are with the Na and the Ga nucleus. These interactions are about 4-5 times stronger than a Te-

Te interaction of the same type. However, if Na is dynamic and the stochastic processes have a high rate the through space diploe-dipole couplings of Na and Te will weaken and eventually become sizable with the Te-Te interactions and at a very high dynamic rate of Na only the Te-Te/Ga dipole interaction remains for linebroadening. The Te-Ga interaction may also be influenced by the probable fast relaxation of Ga as the nucleus has a quadrupolar moment that may lead to fast spin-lattice (T1) relaxation.

S10 Comparison of ^{23}Na NMR of solid-state and sol-gel synthesis of NZTO

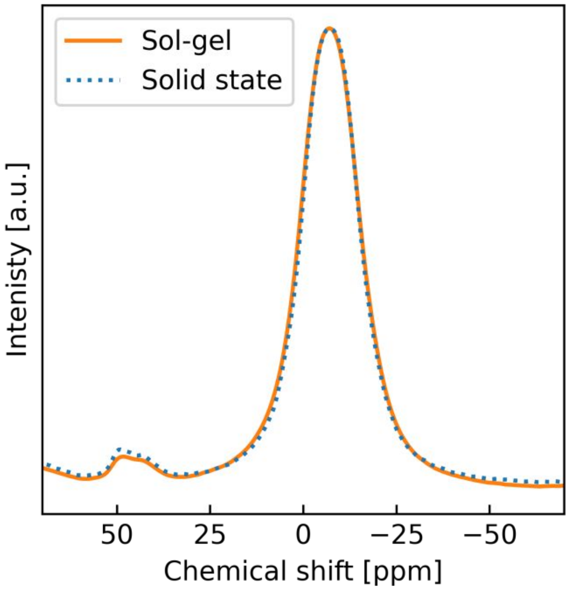

Figure S7: ^{23}Na NMR comparison between NZTO synthesized using sol-gel and solid state synthesis. The similarity of the two spectra demonstrate that while particle morphology and size might be changed by synthesis method, the local structure is clearly similar. There are minor deviations between the spectra, which can either point to very small physical differences from e.g. surfaces or interphases, or be from different phasing of the spectra. The solid-state synthesis was synthesized as reported by Li et al.[12]

References

1 G. Kresse and J. Hafner, Ab initio molecular dynamics for liquid metals, *Phys. Rev. B*, 1993, **47**, 558–561.

2 G. Kresse and J. Furthmüller, Efficiency of ab-initio total energy calculations for metals and semiconductors using a plane-wave basis set, *Comput. Mater. Sci.*, 1996, **6**, 15–50.

3 G. Kresse and J. Furthmüller, Efficient iterative schemes for ab initio total-energy calculations using a plane-wave basis set, *Phys. Rev. B*, 1996, **54**, 11169–11186.

4 G. Kresse and D. Joubert, From ultrasoft pseudopotentials to the projector augmented-wave method, *Phys. Rev. B*, 1999, **59**, 1758–1775.

5 H. J. Monkhorst and J. D. Pack, Special points for Brillouin-zone integrations, *Phys. Rev. B*, 1976, **13**, 5188–5192.

6 C. L. Fu and K. M. Ho, First-principles calculation of the equilibrium ground-state properties of transition metals: Applications to Nb and Mo, *Phys. Rev. B*, 1983, **28**, 5480–5486.

7 P. Pulay, Convergence acceleration of iterative sequences. the case of scf iteration, *Chem. Phys. Lett.*, 1980, **73**, 393–398.

8 A. Katerinopoulou, T. Balic-Zunic and L. F. Lundegaard, Application of the ellipsoid modeling of the average shape of nanosized crystallites in powder diffraction, *J. Appl. Crystallogr.*, 2012, **45**, 22–27.

9 M. A. Evstigneeva, V. B. Nalbandyan, A. A. Petrenko, B. S. Medvedev and A. A. Kataev, A new family of fast sodium ion conductors: Na2M 2TeO6 (M = Ni, Co, Zn, Mg), *Chem. Mater.*, 2011, **23**, 1174–1181.

10 M. Yu and D. R. Trinkle, Accurate and efficient algorithm for Bader charge integration, *J. Chem. Phys.*, 2011, **134**, 064111.

11 F. Bianchini, H. Fjellvåg and P. Vajeeston, Nonhexagonal Na Sublattice Reconstruction in the Super-Ionic Conductor Na 2 Zn 2 TeO 6 : Insights from Ab Initio Molecular Dynamics, *J. Phys. Chem. C*, 2019, **123**, 4654–4663.

12 X. Li, F. Bianchini, J. Wind, P. Vajeeston, D. Wragg and H. Fjellvåg, P2 Type Layered Solid-State Electrolyte Na 2 Zn 2 TeO 6 : Crystal Structure and Stacking Faults , *J. Electrochem. Soc.*, 2019, **166**, A3830–A3837.
